# Class-wide sequence, structural, and docking-profile diversity of insect odorant receptors and structural specialization of Orco

**DOI:** 10.7554/eLife.110877

**Published:** 2026-11-03

**Authors:** Tianmin Zhang, Xuanxiao Yang, Yufang Fu, Wei Xue, Yifeng Zhang, Suyang Duan, Yangming Yin, Yi Guo, Chenxi Gao, Yang Liu, Gang Li, Chang Xu, Huimeng Lu

**Affiliations:** 1 School of Life Science and Technology, Northwestern Polytechnical University Xi'an China; 2 College of Life Sciences, Shaanxi Normal University Xi'an China; CNRS - Universite Paris Cite France; New York University United States

**Keywords:** insect, odorant receptor, similarity network, molecular docking, macroevolution, *D. melanogaster*, Other

## Abstract

Insect olfaction is facilitated by a heterotetrameric odorant receptor–odorant receptor co-receptor (OR-Orco) complex. However, extensive sequence divergence and lineage-specific expansion have complicated large-scale comparisons of OR repertoires across insects. Here, we integrated sequence similarity, structural similarity, and molecular docking to analyze ORs from 115 insect species. OR repertoire size was associated with adult diet, larval diet, and habitat, but not with circadian rhythm. We developed a ‘trunk–branch’ network framework to classify ORs according to sequence and structural similarity and used large-scale molecular docking to derive comparative docking profiles. Despite marked differences in sequence- and structural-community composition, almost all sampled orders contained ORs assigned to all six docking-profile communities. Holometabolous insects showed higher OR sequence-community diversity than non-holometabolous insects, and docking-profile composition differed between extant lineages whose order-level origins predate or postdate the end-Permian mass extinction. Most notably, we identified a highly conserved extracellular β-sheet in Orco that restricts ligand access to the binding pocket and may contribute to its reduced capacity to bind natural odorants. The emergence of Orco represents a key evolutionary transition from homomeric receptors to heteromeric OR–Orco complexes, and our results suggest that this transition may have been accompanied by pronounced specialization of the extracellular domain and binding pocket. Together, these analyses provide a class-wide framework for comparing insect OR diversity and generate testable hypotheses for future experimental and evolutionary studies.

## Introduction

Olfactory systems play a critical role in animal survival strategies, governing critical behaviors from nutrient acquisition to mate selection (Bargmann, 2006; Nei et al., 2008; Kaupp, 2010). Within the kingdom Animalia, insects—the most speciose metazoan group—have evolved exceptionally sophisticated chemosensory systems to interpret complex volatile landscapes (Nei et al., 2008; Kaupp, 2010; Mora et al., 2011; Robertson, 2019). The extraordinary sensitivity of insect olfaction to hydrophobic volatile organic compounds (VOCs) is facilitated by a heterotetrameric channel complex consisting of odorant receptors (ORs) and odorant receptor co-receptors (Orco; Clyne et al., 1999; Vosshall et al., 1999; Sato et al., 2008). Insect ORs represent a distinct protein superfamily that originated independently of vertebrate ORs, with no shared homology between the two (Sato et al., 2008; Hansson and Stensmyr, 2011). Studies have shown that this ancient structural fold has a deeper phylogenetic origin (Benton and Himmel, 2023; Himmel et al., 2023). While vertebrate ORs have been extensively studied (Niimura and Nei, 2005; Hughes et al., 2018; Pacalon et al., 2023; Policarpo et al., 2024), the extremely low sequence similarity among insect ORs has made resolving their internal sequence, structural, and functional differences challenging (Robertson and Wanner, 2006). Extensive studies have reported advances in the olfactory recognition mechanism and OR function (Guo et al., 2021; Chang et al., 2023; Depetris-Chauvin et al., 2023; Jiang et al., 2024), which have greatly facilitated a deeper understanding of insect olfactory perception. However, a class-wide integrative framework linking OR sequence divergence, structural variation, and docking-derived binding-profile diversity is still lacking. Such a framework is needed to distinguish broadly conserved patterns from lineage-specific features and to place species-level OR diversification in a broader macroevolutionary context.

Within the class Insecta, Orcos exhibit exceptional conservation (>400 Mya evolutionary stability), whereas lineage-specific ORs display unprecedented diversity, far exceeding that of vertebrate ORs (Larsson et al., 2004; Nei et al., 2008; Brand et al., 2018; Robertson, 2019; Wicher and Miazzi, 2021). Although several studies have linked OR repertoire evolution to ecological adaptation in specific insect lineages, a unified class-wide comparison remains lacking (Jongepier et al., 2022; Yin et al., 2022; Singh et al., 2025). Traditional phylogeny-driven subfamily categorization, constrained by extreme cross-order sequence divergence, fails to capture the continuum of OR evolutionary innovation (Zhou et al., 2012; Zhou et al., 2015; Mitchell et al., 2020; Legan et al., 2021; Tian et al., 2022; Yin et al., 2022). Unlike vertebrate G-protein-coupled ORs, insect ORs function as ligand-gated ion channels by assembling with the conserved co-receptor Orco, with recent cryo-EM structures supporting a 1:3 OR–Orco heterotetrameric model (Jones et al., 2005; Nei et al., 2008; Kaupp, 2010; Jones et al., 2011; Wang et al., 2024; Zhao et al., 2024). However, the exact in vivo stoichiometry remains to be fully resolved, and alternative arrangements such as 2:2 may also be possible (Wang et al., 2024). The mechanistic drivers of this functional divergence raise key evolutionary questions: This functional specialization raises two key questions: which structural features distinguish Orco from ligand-selective ORs, and how might these features contribute to its reduced capacity to bind natural odorants?

Identifying the binding profiles of ORs for VOCs is fundamental to understanding how insect olfactory systems perceive and adapt to their environments (McBride et al., 2014; Auer et al., 2020). To date, numerous studies have characterized the ligand-binding profiles of various insect ORs using heterologous expression systems (Hallem and Carlson, 2006; Carey et al., 2010; de Fouchier et al., 2017; Guo et al., 2021; Chang et al., 2023; Jiang et al., 2024). However, when attempting to investigate the functional relevance of tens of thousands of ORs across the class Insecta at a macroevolutionary scale, experimental methods are no longer feasible due to their long timeframes and low throughput. For comparative analyses at this scale, computational approaches provide a practical means of generating hypotheses that can subsequently be prioritized for experimental testing. Although machine-learning methods remain constrained by the available training data (Boyle et al., 2013; Chepurwar et al., 2019; Caballero-Vidal et al., 2020), molecular docking offers a scalable complementary approach for comparing potential receptor–ligand interactions across large OR repertoires (Lyu et al., 2019; Comte et al., 2025). This strategy has previously been applied to mammalian OR repertoires: by employing large-scale molecular docking and OR-binding profile prediction, researchers have revealed differences in olfactory perception among bats with different diets, as well as dynamic trade-offs between vision and olfaction in primates (Zhang et al., 2024; Chi et al., 2025). These results suggest that molecular docking can facilitate the exploration of macroevolutionary patterns.

In this study, we characterized large-scale patterns of OR diversification across 115 species spanning 16 insect orders. Using a framework that integrates network analysis, protein structure prediction, and molecular docking, we constructed sequence-similarity (SSN), structural-similarity (StSN), and docking-profile similarity networks (dPSN) to classify ORs from three distinct perspectives. We identified differences in OR sequence-community diversity between holometabolous and non-holometabolous insects, as well as differences in docking-profile community composition among extant lineages whose order-level origins fall on opposite sides of the end-Permian mass extinction (EPME). Across this class-wide sampling, OR repertoire size was associated with adult diet, larval diet, and habitat, but not with circadian rhythm. Moreover, the conserved β-sheet in the extracellular loop 2 (EL2) region of Orco, together with its specialized binding-pocket architecture, is associated with its reduced ligand-binding capacity and represents a prominent structural specialization of the co-receptor lineage.

## Results

### Characterization of the insect odorant receptor gene repertoire

**Figure 1.**
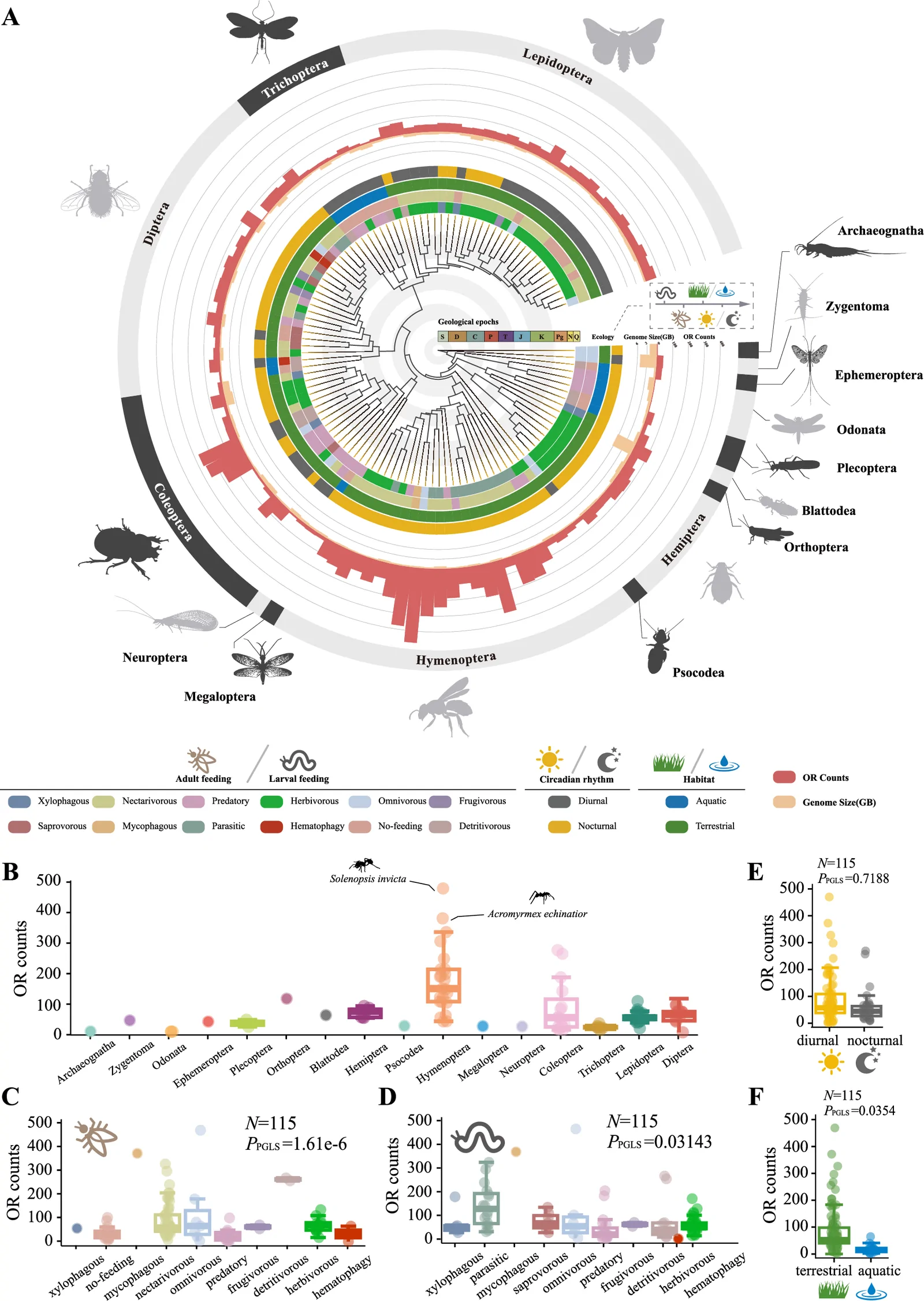
Evolutionary ecology of insect OR repertoires (Figure 1—source data 1–3). (**A**) Phylogeny of 115 insect species (see Materials and methodsfor details). Genome size and the number of OR genes for each species are shown as bar plots. Ecological parameters, including larval and adult diets, habitat, and circadian rhythm, are displayed as labels with color coding corresponding to the legend below. Different geological periods are separated by shaded intervals. The tree was visualized using the iTOL (Letunic and Bork, 2024). (**B**) Boxplot showing the number of OR genes for each insect order. (**C–F**) Results of two-sided pGLS tests assessing the relationship between intact OR gene numbers and various ecological parameters: adult diet (**C**), larval diet (**D**), circadian rhythm (**E**), and habitat (**F**). Dots represent OR counts of each species. N represents the counts of species. Animal silhouettes were obtained from PhyloPic.org. Figure 1—source data 1.Genome information. Figure 1—source data 2.Living habits of 115 species. Figure 1—source data 3.Insect species tree.

We compiled all published insect genomes (as of December 2022), selecting the highest-quality genome assembly (contig N50 >1 Mb) from each family as the representative species. Our study includes 115 species, featuring 8 representative species from different insect orders with previously annotated ORs (Engsontia et al., 2008; Wang et al., 2015; Zhou et al., 2015; Brand et al., 2018; Robertson et al., 2019), spanning 16 orders and 111 families. This approach ensured a comprehensive analysis across the class Insecta (Figure 1A). To increase annotation reliability, we implemented the most stringent protein superfamily member screening strategy to date (Figure 1—figure supplement 1A). A total of 8905 structurally intact ORs were retained for downstream analyses . Benchmarking against curated OR repertoires showed that the automated pipeline recovered most previously reported ORs and that stringent structural filtering produced a high-confidence set of structurally intact receptors (Appendix 1, Supplementary file 1, and Figure 1—figure supplement 1B–C). For most species with comparable genome-based references, retained OR counts exceeded 70% of published values. Our analysis revealed substantial variation in OR counts across insect species. Hymenoptera exhibited the highest number of ORs and accounted for eight of the top 10 species with the largest OR repertoires (Bonasio et al., 2010). In our OR dataset, Odonata had the fewest intact ORs, with a mean of 4 (N=3). Hymenoptera also showed the widest range in OR counts (min: 38, max: 473, N=23), followed by Coleoptera (min: 10, max: 271, N=15, Figure 1B).

**Figure 1—figure supplement 1.**
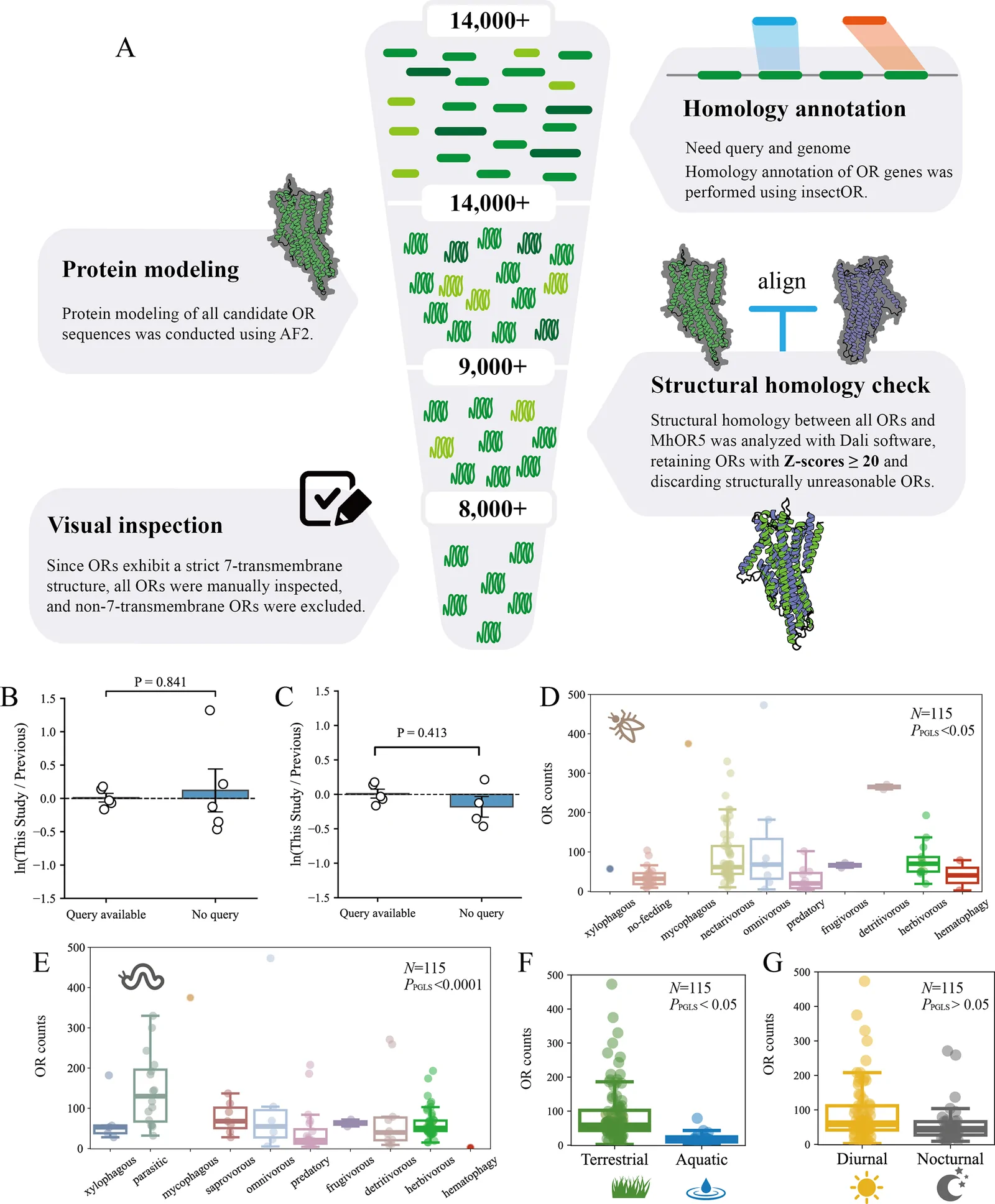
Structural screening strategy and pGLS sensitivity analysis of OR quantity. (**A**) The stringent screening strategy for complete OR genes. All homologously annotated OR genes underwent structure modeling. Structural similarity was used as an indirect measure to assess sequence reliability (Dali Z-score>20). Finally, manual inspection was performed to refine the intact OR genes. For detailed procedures, refer to the Materials and methods. (**B**) Comparison of OR counts from this study and previous annotations between species with and without conspecific sequences in the query set. Error bars represent means ± SEM. Each dot represents one species; 4<n<5. Significance was assessed using the Mann–Whitney U test. (**C**) Comparison after excluding the OR annotation results for the outlier *Anagrus nilaparvatae*. (**D–G**) pGLS tests for adult diet, larval diet, habitat, and circadian rhythm after OR counts were updated to the larger of structurally intact OR counts and published OR counts.

Previous studies have shown that in certain insect orders and specific lineages, OR repertoire characteristics are associated with various ecological traits (McBride, 2007; Liu et al., 2021; Schrader et al., 2021; Jongepier et al., 2022; Singh et al., 2025). To determine whether these relationships extend to the class Insecta, we analyzed four ecological factors: adult diet, larval diet, habitat, and circadian rhythm. Adult and larval diets represent stage-specific ecological traits, and our analysis of OR gene numbers at the genomic level aimed to reveal their relationship with long-term evolutionary pressures shaping OR gene family size. Our results revealed a significant correlation between insect OR counts and dietary preferences, both in adults (Figure 1C, p=1.61 × 10⁻⁶) and larvae (Figure 1D, p=0.03143). Habitat was also significantly associated with OR counts (p=0.0354), with terrestrial insects exhibiting more ORs than aquatic insects do (Figure 1F). However, there was no significant correlation between circadian rhythm and OR count (p=0.7188, Figure 1E). Overall, our findings suggest that insect OR counts correlate with dietary specialization (in both larvae and adults) and habitat type but are not associated with circadian rhythm. We then repeated the phylogenetic generalized least squares (pGLS) analyses using the larger of our structurally intact OR count and the published OR count, and the main ecological associations were retained (Figure 1—figure supplement 1D-G). This indicates that the main OR-count ecological analyses are reasonably robust to moderate underestimation caused by missed ORs or strict structural filtering.

### OR sequence similarity network

**Figure 2.**
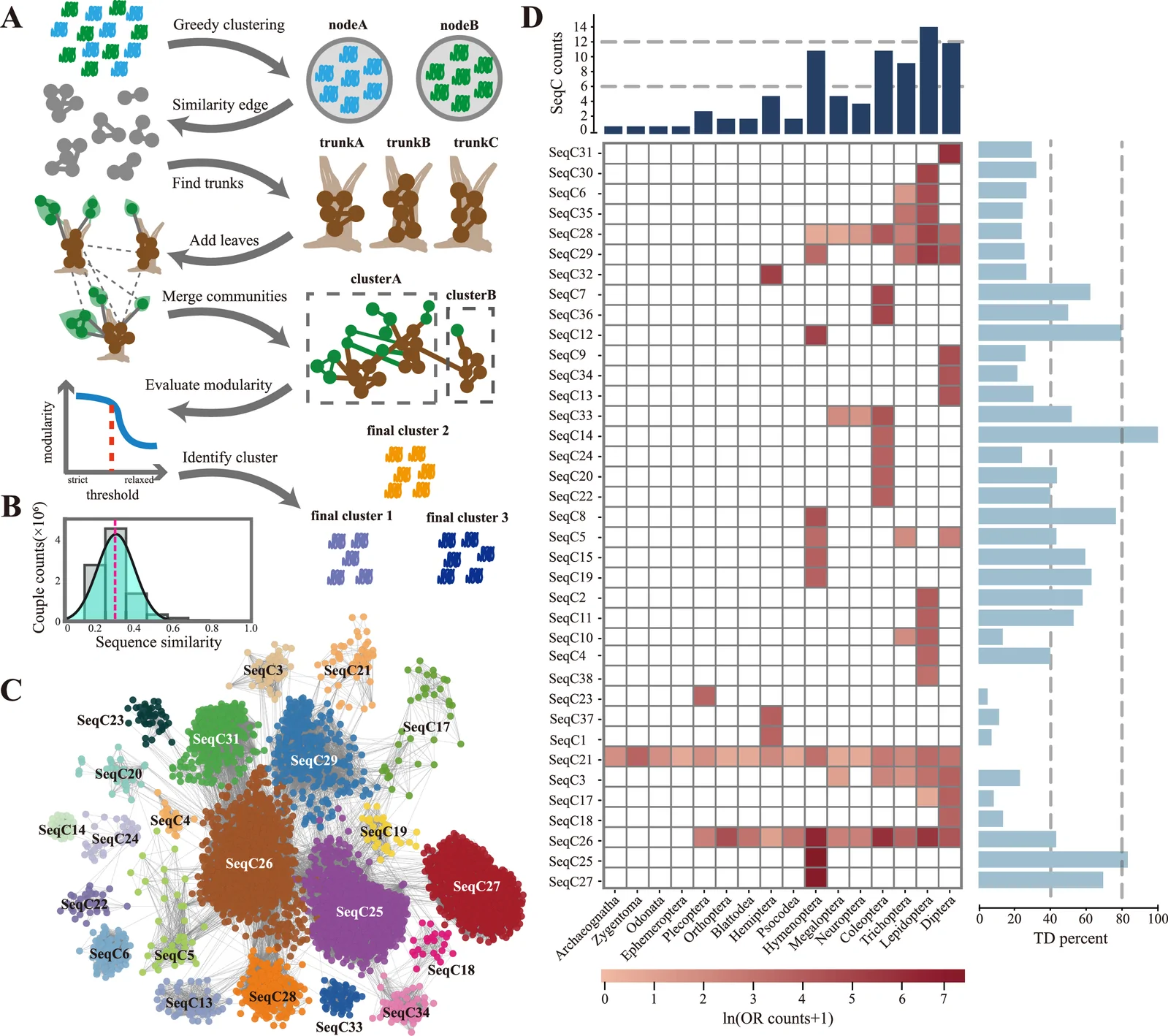
Subfamily classification of ORs based on SSN (Figure 2—source data 1 and Figure 2—source data 2). (**A**) Schematic representation of the ‘trunk–branch’ strategy pipeline for constructing the similarity network and classifying subfamilies. First, all ORs were subjected to greedy clustering based on sequence similarity to generate nodes. The average similarity between nodes was then calculated, and nodes were treated as units to identify core regions (trunks) using a strict similarity threshold. By gradually relaxing the threshold, new core regions and additional nodes (branches) emerged. After a final merging step based on defined criteria, the optimal community structure was determined using modularity as an index (see Materials and methods). (**B**) Distribution of pairwise sequence similarity among all ORs. The red line indicates the mean similarity. (**C**) SSN of ORs constructed using the ‘trunk–branch’ strategy. Each node represents ORs sharing >60% sequence identity. Edges represent node pairs with an average sequence alignment E-value of <1 × 10⁻²⁵. Node colors indicate SeqC classifications. (**D**) Heatmap depicting the number of ORs within different SeqCs across various insect orders. Due to substantial differences in OR numbers among orders, the values are ln(count +1)-transformed. The bar plot above the heatmap shows the number of SeqCs present in each order, while the bar plot on the right indicates the proportion of ORs within each SeqC generated by tandem duplication. Two ORs located within 5 kb of each other on the genome were defined as tandem duplicates. TD, tandem duplication. Figure 2—source data 1.Amino acid sequences of insect ORs. Figure 2—source data 2.Orthologous groups identified within SeqC26, SeqC28, and StrC23.

This study includes species representing the full phylogenetic diversity of the class Insecta, and their OR sequences exhibit substantial variation in amino acid similarity (min = 13.7%, max = 97.6%, and mean = 29.6%; Figure 2B). Because of the extensive sequence divergence among ORs from different insect orders, deep relationships are difficult to resolve reliably in a single class-wide phylogeny. We therefore constructed an SSN based on all-vs-all sequence comparisons as a complementary approach to characterize large-scale patterns of OR sequence similarity. In the SSN, each node represents a cluster of ORs sharing at least 60% sequence similarity, and edges are drawn between nodes if the average alignment E value <1e-25 (Figure 2—figure supplement 1A). Nodes with a greater number of connections indicate closer evolutionary relationships.

**Figure 2—figure supplement 1.**
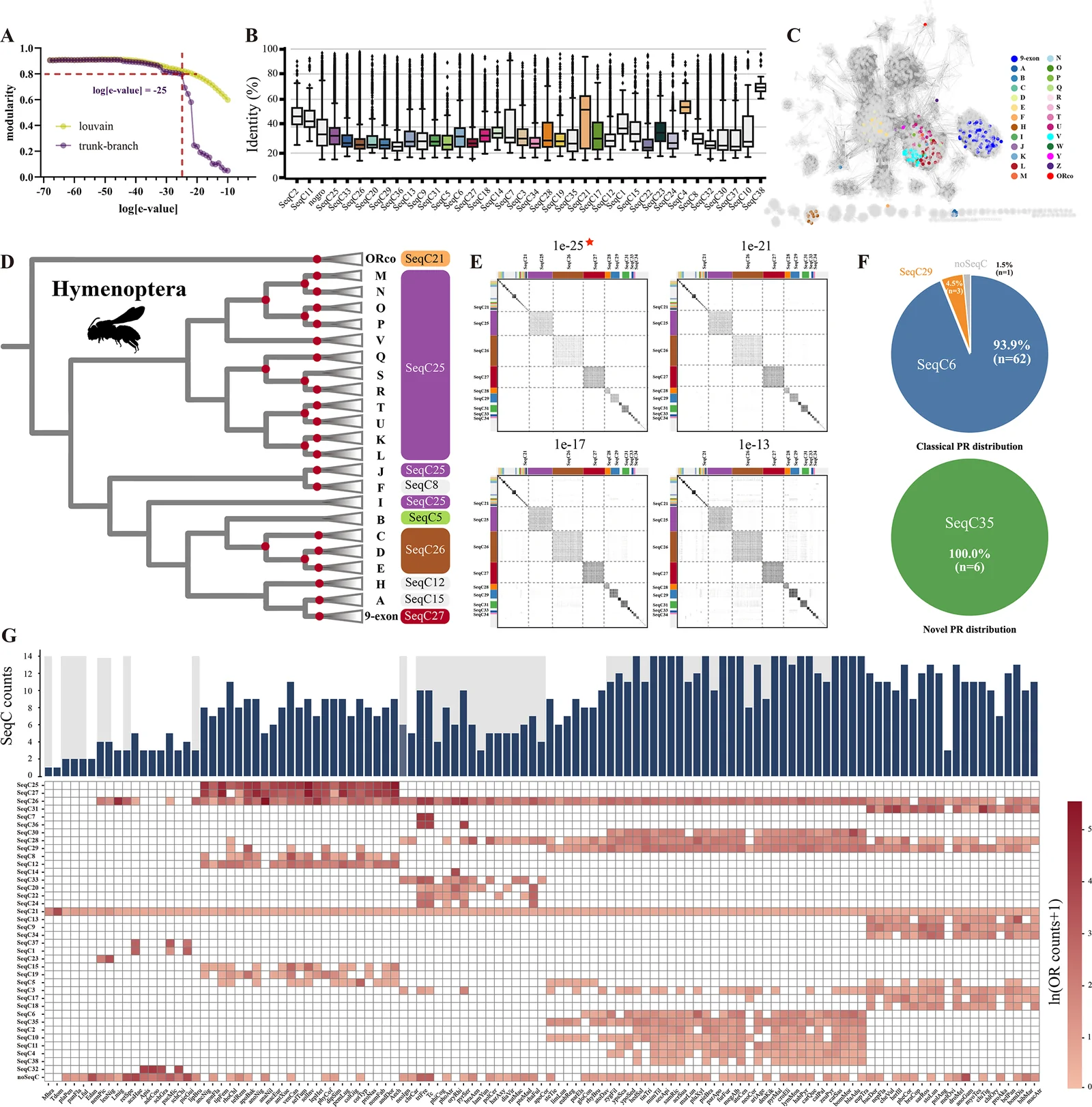
SSN intra-subfamily OR sequence similarity and evidence supporting the accuracy of SeqC. (**A**) Modularity changes between SeqCs at different SSN alignment e-value thresholds. Purple represents modularity obtained using the ‘trunk–branch’ algorithm, while yellow denotes the theoretical optimal modularity based on the Louvain algorithm. (**B**) Pairwise sequence similarity within different SeqCs. The SeqC colors are consistent with Figure 2C, while gray indicates SeqCs with no interactions with others. Boxplots represent the first quartile minus 1.5 times the interquartile range, the interquartile range, the first quartile, the mean, the third quartile, and the third quartile + 1.5 interquartile range. Dots indicate outliers. (**C**) Mapping of *Acromyrmex echinatior* (Aech) OR subfamilies onto the SSN. Different colors represent previously defined hymenopteran subfamilies. (**D**) Phylogenetic relationship between previously defined hymenopteran subfamilies and our SeqC classification. Red dots indicate branches with bootstrap values greater than 80. Branches with the same subfamily designation as previous studies are collapsed. The phylogenetic tree is based on the previous study (Zhou et al., 2015). Animal silhouettes were obtained from PhyloPic.org. (**E**) Greyscale interaction matrix showing interactions between SeqCs under different e-value thresholds. At an e-value of 1e-25, SeqC boundaries are distinct. As the threshold increases to 1e-17, interactions between SeqCs become more frequent, with extensive interactions observed at 1e-13. The SeqC divisions remain clear within approximately 8 orders of magnitude. Different color bars represent different SeqCs, consistent with Figure 2C. (**F**) Proportions of Lepidopteran PRs distributed across different SeqCs. (**G**). Heatmap showing the number of ORs within different SeqCs across species. Due to significant variation in OR numbers among species, the OR counts are ln-transformed. The top bar plot represents the number of SeqC types in each species. Gray shading indicates boundaries between insect orders.

Using the ‘trunk–branch’ strategy (Figure 2A), we identified 37 OR sequence communities (SeqCs), which together account for 94.9% of all ORs . Among them, 22 SeqCs demonstrated clear interaction relationships (Figure 2C). The sequence similarity within each SeqC was predominantly between 20% and 30% (Figure 2—figure supplement 1B). By mapping previously classified *Acromyrmex echinatior* sequences using phylogenetic methods (Zhou et al., 2015), we found that most subfamilies—such as 9-exon, Orco, F, H, B, and A—were independently delineated (Figure 2—figure supplement 1C) and exhibited well-defined boundaries (Figure 2—figure supplement 1E). Our classification did not disrupt any known subfamilies and instead merged some families with minor differences (Figure 2—figure supplement 1, C and D). This comparison indicates that SSN-based communities are broadly consistent with established phylogenetic subfamilies, while capturing sequence-similarity modules rather than strictly monophyletic clades. The SSN is not intended to replace phylogenetic reconstruction, but rather to provide a complementary framework for summarizing sequence relationships among thousands of highly divergent ORs at a class-wide scale. It allows major patterns of OR sequence similarity and diversification to be visualized more clearly across the entire dataset. Phylogenetic analyses can then be used to further resolve evolutionary relationships within specific sequence communities. We further evaluated the SSN using curated lepidopteran pheromone receptors (PRs). To distinguish different PR lineages, experimentally validated classic PRs and the recently reported new PR lineage were analyzed separately (Bastin-Héline et al., 2019). Classic PRs were predominantly assigned to SeqC6 (93.9%), whereas the new PR lineage was assigned to SeqC35 (Figure 2—figure supplement 1F), supporting the hypothesis of multiple independent origins of PRs (Bastin-Héline et al., 2019). These results further support the applicability and robustness of SSN-based community partitioning.

SeqC21 includes the Orco group as well as all ORs from *Thermobia domestica* and *Machilis hrabei* (Figure 2—figure supplement 1G), suggesting that ORs in early wingless insects share high sequence similarity with Orco (Brand et al., 2018). In addition to SeqC21, most SeqCs are order-specific, indicating substantial sequence divergence among ORs from different insect orders (Figure 2D). SeqC26 and SeqC28 are large shared clusters that may contain cross-order orthologous relationships. In SeqC26, we identified 55 orthologous groups, four of which spanned multiple insect orders . OGG54 included ORs from Orthoptera, Blattodea, Trichoptera, and Hymenoptera. This group contains the locust receptors LmigOR5 and LmigOR4. LmigOR5 has been reported to bind geranyl acetone and to be associated with avoidance behavior in the migratory locust (Chang et al., 2023). The functions of the corresponding receptors in other insect orders remain to be tested, and we now present these as candidate conserved modules rather than confirmed functional orthologs. In contrast, we did not identify clear cross-order orthologous groups in SeqC28.

Lepidoptera was found to contain the greatest number of SeqCs, reflecting the highest level of OR sequence divergence. Additionally, we identified SeqC25 and SeqC27 as two exceptionally large, Hymenoptera-specific groups, comprising 39.8% and 31.9% of all Hymenopteran ORs, respectively. SeqC27 corresponds to the previously identified Hymenopteran 9-exon family (Figure 2—figure supplement 1C and D), which originated through tandem duplication events (Engsontia et al., 2015; Zhou et al., 2015; McKenzie et al., 2016; Legan et al., 2021). Interestingly, we found that SeqC25 exhibited an even higher rate of tandem duplication than did SeqC27, suggesting that SeqC25 also expanded through extensive tandem duplication. Notably, among SeqCs with tandem duplication rates exceeding 60%, five of seven are exclusive to Hymenoptera, indicating that OR lineages in this order have undergone particularly intense tandem duplication expansion compared with other insect orders (Legan et al., 2021).

### Structural diversity of insect ORs

**Figure 3.**
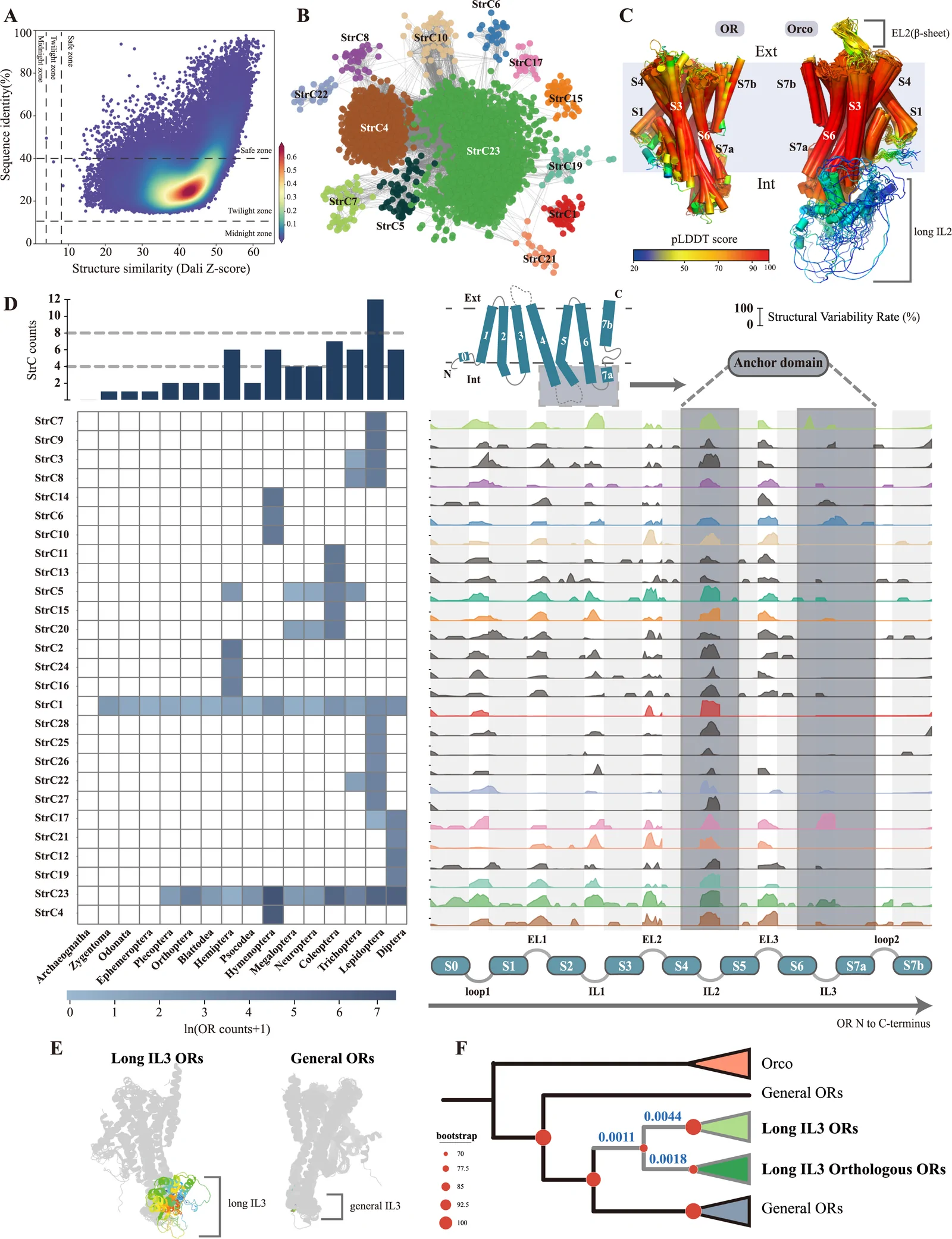
StSN and structural community diversity of ORs (Figure 3—source data 1). (**A**) Sequence similarity versus structural similarity of insect ORs, assessed using pairwise comparisons with Dali. Each dot represents comparison between a pair of ORs, with color indicating point density. (**B**) StSN of ORs constructed using the ‘trunk–branch’ strategy. Nodes represent ORs with pairwise Dali Z-scores >50. Edges connect nodes with an average pairwise Z-score of at least 42.9. Node colors denote StrCs classifications. (**C**) Structural comparison of ORs and Orco. The structures were aligned using US-align, with multiple structural alignments performed for annotated Orco and OR structures (visualized in PyMol). Colors represent pLDDT scores provided by AlphaFold2, indicating the confidence of structural modeling. (**D**) Visualization of differences among StrCs. The heatmap shows the number of ORs within different StrC across insect orders, with OR counts ln(count +1)-transformed due to large inter-order variations. The bar plot above the heatmap indicates the number of StrC in each order. On the right, a ridge plot illustrates the flexibility rates of different structural regions across StrCs. Flexibility rate = 1 - conservation rate. Light gray represents transmembrane regions, white denotes loop regions, and dark gray marks the anchor domain of ORs. Colors correspond to StrCs, with gray indicating StrCs without interactions. (**E**) Two types of ORs in StrC17—long IL3 ORs and general ORs—with the IL3 regions highlighted in color. (**F**) Phylogenetic analysis of StrC17 ORs based on amino acid sequences. Red circles indicate bootstrap values, with most ORs collapsed based on bootstrap support. Gray branches represent the clade of long IL3 ORs, and the dN/dS values are shown in blue on the corresponding evolutionary branches. Figure 3—source data 1.The likelihood ratio test results for StrC17 ORs.

Insect ORs fall within the ‘twilight zone’ (10–40% sequence identity) of sequence alignment. However, nearly all ORs remain within the ‘safe zone’ in structural alignment (Figure 3A).

**Figure 3—figure supplement 1.**
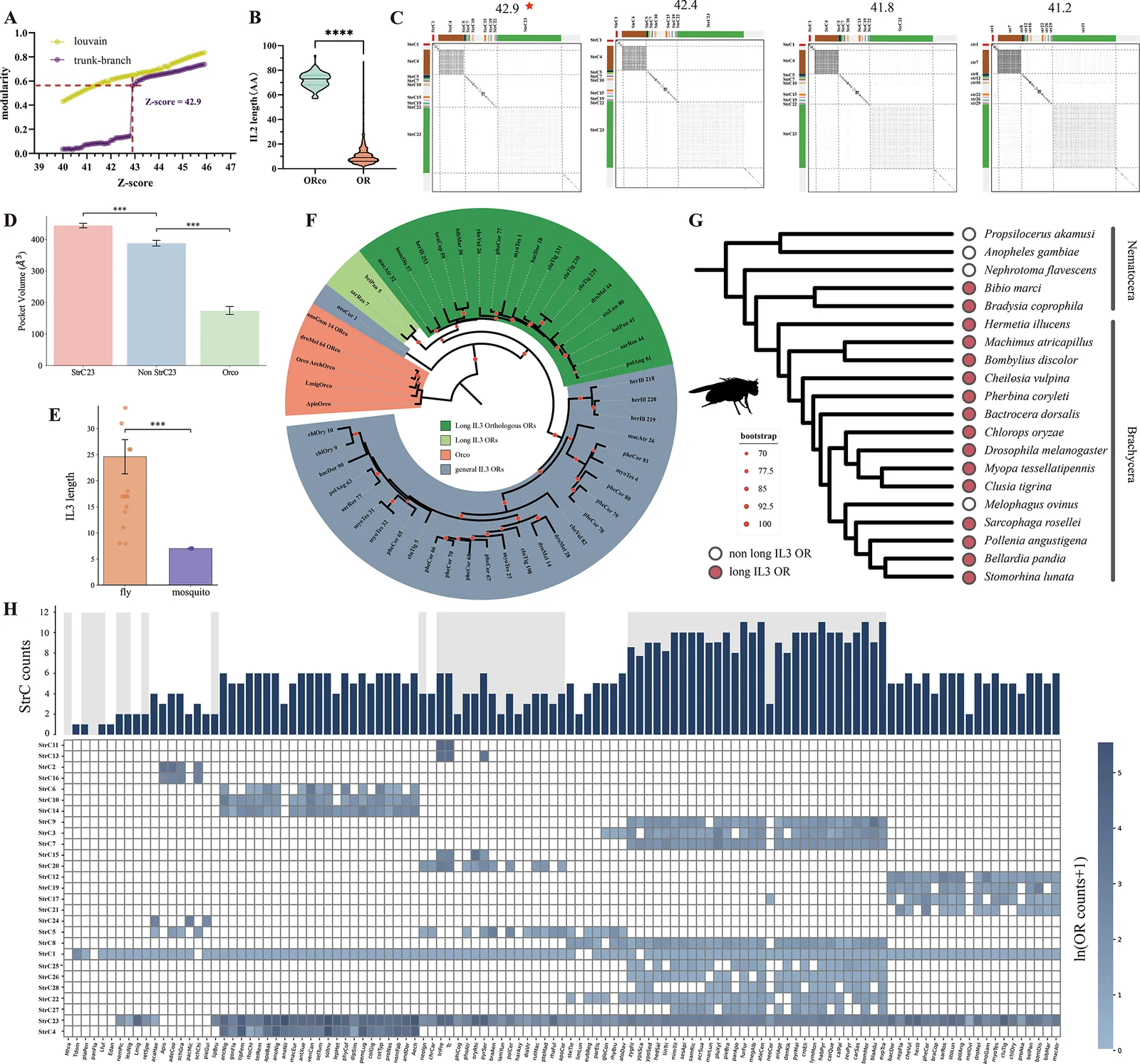
StrC classification and the phylogenetic tree of StrC17 ORs. (**A**) Modularity changes between StrCs at different Dali Z-score thresholds. Purple represents modularity obtained using the ‘trunk–branch’ algorithm, while yellow denotes the theoretical optimal modularity based on the Louvain algorithm. (**B**) Comparison of IL2 loop amino acid lengths between all ORs (n = 8791) and Orco (n=114). A two-sided t-test was used to assess significance. (**C**) Grayscale interaction matrix showing interactions between StrCs under different Z-score thresholds. At a Z-score of 42.9, the boundaries between StrCs are distinct. At a threshold of 41.8, interactions between StrCs increase, with extensive interactions observed at 41.2. Different color bars represent distinct StrCs, consistent with Figure 3B. Gray indicates StrCs without interactions. (**D**) Binding-pocket volume of the shared structural cluster StrC23. Error bars represent means ± SEM; n = 4124, 4667, and 114 receptors for StrC23, non-StrC23, and Orco, respectively.Tukey’s post hoc test was performed after one-way ANOVA. (**E**) Comparison of IL3 length between the conserved fly OR cluster that recognizes 1-octen-3-ol and mosquito ORs that recognize 1-octen-3-ol. Error bars represent means ± SEM. Each dot represents one species; n = 20 and 4 for flies and mosquitoes, respectively. Welch’s t-test was used. ***p<0.001. (**F**) Maximum likelihood phylogenetic tree of StrC17 ORs, with Orco as the outgroup. Green indicates long IL3 ORs, and red circles mark bootstrap values. The tree was visualized using iTOL. (**G**) Species tree of dipteran species included in the study. Red circles represent species with long IL3 ORs, while hollow circles indicate species without long IL3 ORs. Animal silhouettes were obtained from PhyloPic.org. (**H**) Heatmap showing the number of ORs within different StrCs across species. Due to significant variation in OR numbers among species, the OR counts are ln-transformed. The top bar plot indicates the number of StrC types in each species. Shading marks the boundaries between insect orders. ****p<0.0001.

To investigate the structural diversity of insect ORs, we constructed a StSN, where each node represents ORs with Z score >50 (from Dali; Holm, 2022). Edges were drawn between nodes if their average Z score was >42.9 (Figure 3—figure supplement 1A). Similar to the SSN, we classified 27 structural communities (StrCs), which together accounted for 87.0% of all ORs . Among these, 13 StrCs exhibited distinct interaction relationships (Figure 3B). The boundaries between StrCs were well defined (Figure 3—figure supplement 1C).

Most StrCs were also order-specific (Figure 3D). StrC1 represents the Orco group, which includes 8 *T. domestica* ORs. These ORs were previously identified as Orco-like genes through sequence analysis (Brand et al., 2018). Compared to ORs, Orco is characterized by two distinct structural features: a β-sheet in EL2 and a long loop in intracellular loop 2 (IL2; Figure 3C, Figure 3—figure supplement 1B). Lepidoptera presented the highest diversity of StrC types (Figure 3D). We found that the transmembrane regions in different StrCs presented low variability, whereas the loop regions presented distinct variable patterns (Figure 3D). In both ORs and Orcos, the IL2 region was identified as the most variable region and the only variable segment within the anchor domain. This high degree of variability may be associated with its role in stabilizing and regulating the anchoring domain (Bahk and Jones, 2016; Jain et al., 2021; Wang et al., 2024; Zhao et al., 2024).

StrC23 is the only structural community present in 12 insect orders and accounts for 46.3% of all structurally intact ORs in the dataset. We found that StrC23 has a much larger binding-pocket volume than other ORs and Orco. From a structural perspective, this suggests that StrC23 receptors may have greater potential to accommodate diverse VOCs (Figure 3—figure supplement 1D). To further investigate the evolutionary basis of this broadly shared structural feature, we examined the phylogenetic and orthology relationships among StrC23 receptors. StrC23 was divided into 156 orthologous groups, 24 of which contained ORs from more than 10 species. However, only a few of these larger groups spanned multiple insect orders: one included ORs from Hymenoptera, Diptera, and Trichoptera, while two included ORs from both Lepidoptera and Trichoptera. Thus, StrC23 does not represent a single conserved cross-order orthologous lineage but instead comprises multiple phylogenetically distinct OR lineages. Its shared large-pocket architecture therefore cannot be explained solely by conservation of a single orthologous lineage and may reflect either structural convergence or retention of an ancient structural feature followed by substantial sequence divergence.

Notably, StrC17 exhibited a high degree of flexibility in the IL3 region. We observed that a subset of ORs within this group possessed unusually long IL3 regions (Figure 3E). These ORs were distributed across 16 families from two suborders of Diptera but were absent from the earliest-diverging families in the Nematocera suborder, including Culicidae, Chironomidae, and Tipulidae (Figure 3—figure supplement 1G and H). After manual inspection, annotation errors were ruled out. The construction of a maximum likelihood tree for StrC17 ORs revealed that ORs with long IL3 regions formed a distinct clade, with one subclade identified as orthologous (Figure 3—figure supplement 1F). To investigate the evolutionary pressures acting on these ORs, we calculated the dN/dS ratios for different branches (Figure 3F). The results revealed that all StrC17 ORs were subject to strong purifying selection (dN/dS = 0.17). The amino acid substitution rate of ORs with long IL3 regions was two orders of magnitude lower than that of other ORs (dN/dS = 0.001–0.004), indicating intensified purifying selection.

### Structural specialization of Orco

**Figure 4.**
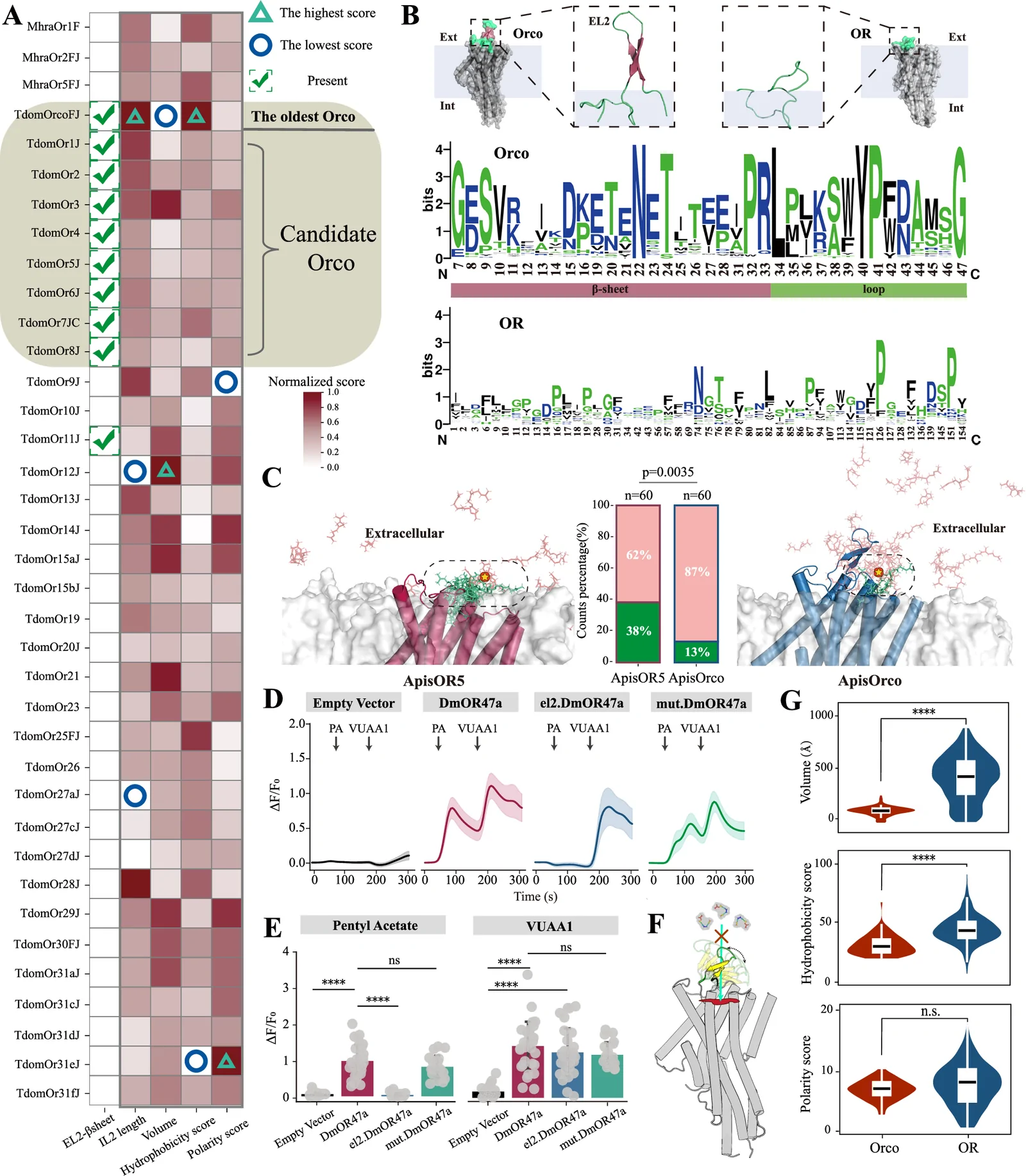
Structural features associated with Orco specialization (Figure 4—source data 1 and 2). (**A**) Differences in EL2, IL2, and binding pocket properties between *M. hrabei* and *T. domestica* ORs. Only ORs with closed binding pockets were analyzed, and all scores were normalized. Circles and triangles represent the minimum and maximum scores after column normalization, respectively. The presence of an EL2 β-sheet is indicated with a check mark. (**B**) Differences in amino acid properties of the EL2 region between OR and Orco. Hydrophilic amino acids are shown in blue, neutral amino acids in green, and hydrophobic amino acids in black (visualized by WebLogo Crooks et al., 2004). The x-axis numbers represent amino acid positions from the multiple sequence alignment, with highly gapped positions removed. (**C**) MD simulation-based analysis of the movement trajectory of VOC (geranyl acetate) near Orco and OR. The red protein represents ApisOR5, and the blue protein represents ApisOrco. The green area indicates the probability of VOC remaining above the binding pocket, while the pink represents its probability of occupying other regions of the protein (see Materials and methods). Statistical significance was evaluated using a Chi-Squared test. The red pentagram indicates the initial position of geranyl acetate in the simulation. (**D**) Changes in relative calcium fluorescence intensity (ΔF/F0) in cells. Cells were stimulated with 100 μM of the OR47a agonist pentyl acetate (PA) at 50  s and 100 μM the Orco agonist VUAA1 at 180 s. el2.DmOR47a: OR47a mutant in which the EL2 region of OR47a was replaced with that of Orco. mut.DmOr47a: the EL2 region of wild-type DmOr47a contains an inserted short peptide. The total number of cells (n=30) was derived from three independent replicate experiments. The shading indicates the 95% confidence interval. (**E**) Relative calcium fluorescence intensity in cells following stimulation with PA and VUAA1, respectively. Error bars represent means ± SEM. n=30. Statistical significance was determined using Tukey’s HSD test. (**F**) Schematic illustration of the mechanism by which the β-sheet structure in the EL2 region of Orco influences VOC proximity to the binding pocket. (**G**) Comparative analysis of binding pocket parameters (volume, hydrophobicity score, and polarity score) between Orco and ORs across all studied insect species. Statistical significance was determined using a two-sided t-test. **** p<0.0001; n.s., not significant. Figure 4—source data 1.Calcium fluorescence responses in cells transfected with each plasmid construct. Figure 4—source data 2.Structural and binding-pocket parameters of MhORs and TdomORs.

Orco evolved from ORs in the common ancestor of Zygentoma and Pterygota (Brand et al., 2018; Thoma et al., 2019). The evolutionary drivers underlying Orco specialization remain unresolved. With the currently available early-diverging insect genomes, we could not reliably identify direct orthologous relationships among early ORs, which limits the reconstruction of ancestral OR states. The *T. domestica* ORs TdomOR1-8 are classified within Orco communities (SeqC21 and StrC1) and occupy a transitional phylogenetic position between ORs and characterized Orcos, representing the closest ORs related to Orcos (Brand et al., 2018). Because they are the extant ORs most similar to Orco in sequence, structure, and phylogenetic position, we used them as comparative proxies for candidate Orco. Comparisons between TdomOrcoFJ and the other TdomOR1–8 receptors may therefore identify structural features potentially associated with Orco recruitment and specialization in the common ancestor of Zygentoma and Pterygota. We found that TdomOrcoFJ has the smallest, least polar, and most hydrophobic binding pocket among TdomOR1-8 (Figure 4A). Molecular docking analysis revealed that TdomOrcoFJ is less suited for VOC binding than other candidates are (Figure 4—figure supplement 1A). These findings suggest that binding-pocket specialization may have been one of the structural factors associated with the evolutionary recruitment of Orco.

**Figure 4—figure supplement 1.**
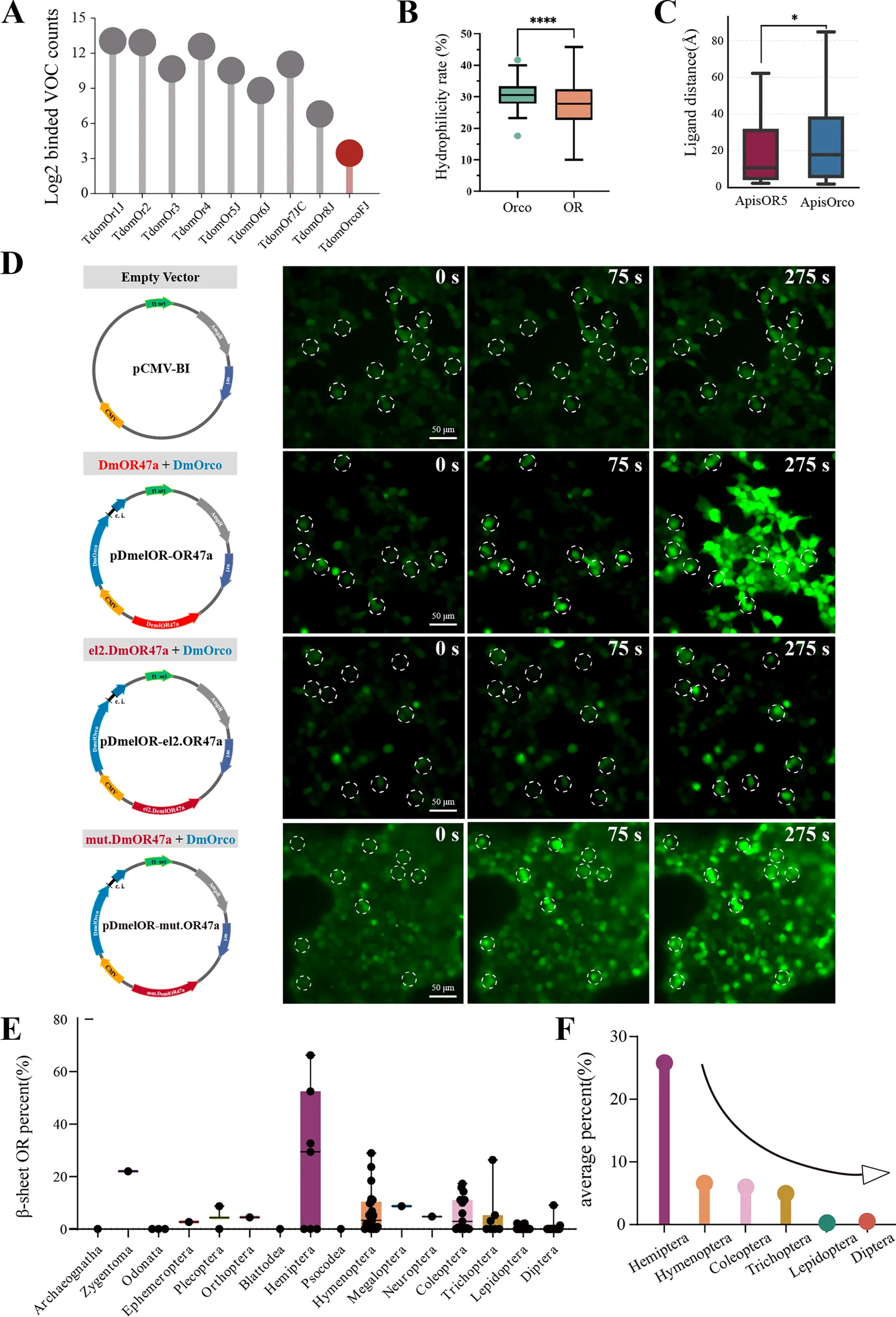
The driving factors underlying the origin of Orco. (**A**) The number of VOCs recognized by Tdom1-8 and Orco. VOCs with a DOCK score < –8 kcal/mol were considered bound. The number of bound VOCs is shown on a log2 scale. (**B**) Proportion of hydrophilic amino acids in the EL2 region of Orco and OR. Statistical significance was assessed using a two-sided t-test. Dots represent outliers. (**C**) The distance between geranyl acetate and its initial position above ApisOR5 and ApisOrco was measured after MD simulation. The results indicate that Orco exhibits a dispersive effect on small molecules. (**D**) Examples of calcium imaging experiments with HEK293 cells co-transfected with DmOr47a+Orco constructs, the el2.DmOr47a mutant construct (in which the EL2 region of DmOr47a was replaced with that of Orco), and the mut.DmOr47a mutant construct (in which a short peptide is inserted into the EL2 region of DmOr47a at the position corresponding to the EL2 structure of Orco, rather than forming a β-sheet structure). Schematic diagrams of different plasmid constructs are shown on the far left. CMV: cytomegalovirus promoter. c.i., chimeric intron; ori, origin of replication. Fluorescence changes are shown following stimulation with PA (middle column) and VUAA1 (right column). White dashed circles indicate the cells used for fluorescence quantification. The experiment was independently replicated three times; results from one representative trial are shown here.Scale bars, 50 μm; the same scale applies to all micrographs in panel D. (**E**) Box plot showing the proportion of EL2 β-sheet ORs among total ORs for different insect orders. Dots represent the proportion of β-sheet ORs in each species. (**F**) Average proportion of β-sheet ORs among total ORs for six major insect orders. The proportion of β-sheet ORs decreases progressively with evolutionary time. * p<0.05, **** p<0.0001.

**Figure 4—video 1.** Molecular dynamics trajectories of VOCs above the binding pockets of OR and Orco.

The reasons behind the loss of Orco function remain unclear, although its conserved binding pocket may act as a molecular sieve (Pacalon et al., 2023). We found that *M. hrabei* ORs lack the EL2 β-sheet but have a long IL2 loop region. Similarly, most *T. domestica* ORs also have a long IL2 loop region, whereas only TdomOR1-8 retains the EL2 β-sheet (Figure 4A). The long IL2 loop was present in ORs from early-diverging insects as well as in candidate Orco and therefore does not provide a distinguishing structural criterion for candidate Orcos. In contrast, the EL2 β-sheet was consistently present in TdomOR1–8 and highly conserved across characterized Orcos. This distribution suggests that the EL2 β-sheet may have emerged as a key structural innovation during Orco specialization and subsequently become conserved in the Orco lineage.

The formation of the EL2 β-sheet may be associated with the loss of Orco function. We observed that the EL2 region of Orco is distinctly divided into two parts: the β-sheet region and the transmembrane loop region (Figure 4B). In the β-sheet region, the proportion of hydrophilic amino acids is 47.8%, whereas it is 14.2% in the transmembrane loop region and 18% in ORs. Overall, across all the insects, the β-sheet region of Orco EL2 presented a significantly greater proportion of hydrophilic amino acids than did the ORs (Figure 4—figure supplement 1B), facilitating its stable presence in the extracellular aqueous environment. The extracellular port is the main pathway through which ligands enter the binding pocket (Renthal and Chen, 2022; Pacalon et al., 2023). Using molecular dynamics (MD) simulations to analyze VOC movement outside the membrane, we found that the presence of the β-sheet in the EL2 region significantly reduced the likelihood of VOCs reaching the binding pocket (Figure 4C, Figure 4—figure supplement 1C and Figure 4—video 1). Using *Drosophila* OR47a as an example, we demonstrated through calcium imaging experiments that replacing the EL2 region of OR47a with that of Orco resulted in a mutant OR47a (el2. DmOR47a) that exhibited markedly reduced calcium responses to pentyl acetate (PA; Figure 4D and E and Figure 4—figure supplement 1D). Additionally, we inserted a non-β-sheet short peptide into the EL2 region of OR47a, and the results showed that its function remained unaffected (Figure 4D and E and Figure 4—figure supplement 1D). This finding suggests that the β-sheet acts as a spatial barrier, limiting VOC access to the binding pocket as a primary physical gate (Figure 4F). Furthermore, we observed that Orco has a smaller and less hydrophobic binding pocket than ORs do (Figure 4G). These characteristics are unfavorable for VOC entry into the binding pocket, representing the key chemical factors contributing to Orco’s loss of natural VOC-binding function.

### Classification of ORs based on docking-profile similarity

**Figure 5.**
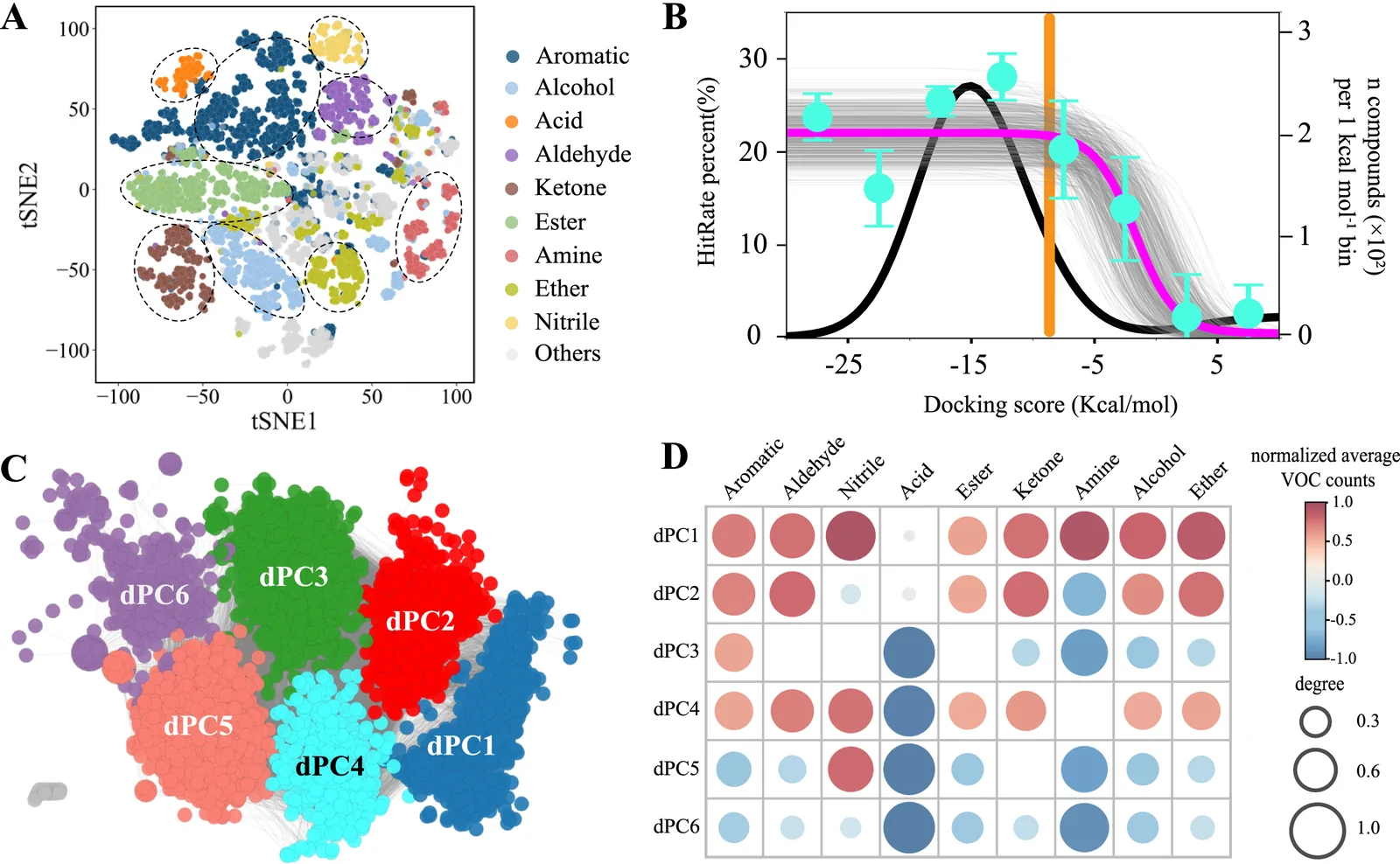
Classification of insect OR docking-profile communities (Figure 5—source data 1 and 2). (**A**) 2D odor space based on molecule physicochemical properties. Each dot represents an odor molecule, with different colors indicating different functional groups. (**B**) Relationship between hit rate and docking score, inferred using Bayesian statistics. The black curve shows the distribution of hit rates and docking scores across all OR–VOC docking results. The top plateau has a hit rate of 22%, while the bottom plateau has a hit rate of 0%. Cyan dots represent the mean hit rate ± SEM for each docking score interval. n = 3.The orange line indicates the peak hit rate at a docking score of –8 kcal/mol. The gray curve represents posterior distributions from Bayesian inference, with n=500. (**C**) dPSN and dPC of ORs. Nodes represent ORs with pairwise PCCs >0.6 based on docking scores. Edges represent node pairs with an average PCC ≥0.48. (**D**) Labels for the recognition of different functional group small molecules by each dPC. Red indicates a tendency to recognize a higher number of odor molecules from a particular functional group, while blue indicates a tendency to recognize fewer. Circle size represents the degree of this tendency. Figure 5—source data 1.The physicochemical properties and functional group labels of the 14,499 VOCs. Figure 5—source data 2.Matrix of all OR–VOC docking scores.

Currently, molecular docking and virtual screening have been successfully applied to the high-throughput prediction of OR binding profiles in mammals, facilitating the exploration of macroevolutionary patterns in predicted olfactory recognition (Zhang et al., 2024; Chi et al., 2025). To better compare the potential binding profiles of insect OR repertoires and classify them accordingly, we collected 14,499 VOCs from four molecular libraries, including pheromones (Li et al., 2023), fragrances (thegoodscentscompany.com), food odors (Chi et al., 2025), and theoretical odors (Mayhew et al., 2022). These VOCs were categorized into nine functional groups (Figure 5A). Although terpenoids are important in insect chemical ecology, they are distributed across multiple regions of odor space (Figure 5—figure supplement 1C). To reduce the docking instability that could result from excessive VOC subdivision, we did not treat terpenoids as a category parallel to the nine basic functional-group categories. We used this comprehensive VOC library to systematically evaluate the docking-derived binding profiles of ORs, aiming to characterize the potential ligand-binding diversity encoded by insect OR repertoires, even though some of these compounds may never be encountered by insects in nature.

**Figure 5—figure supplement 1.**
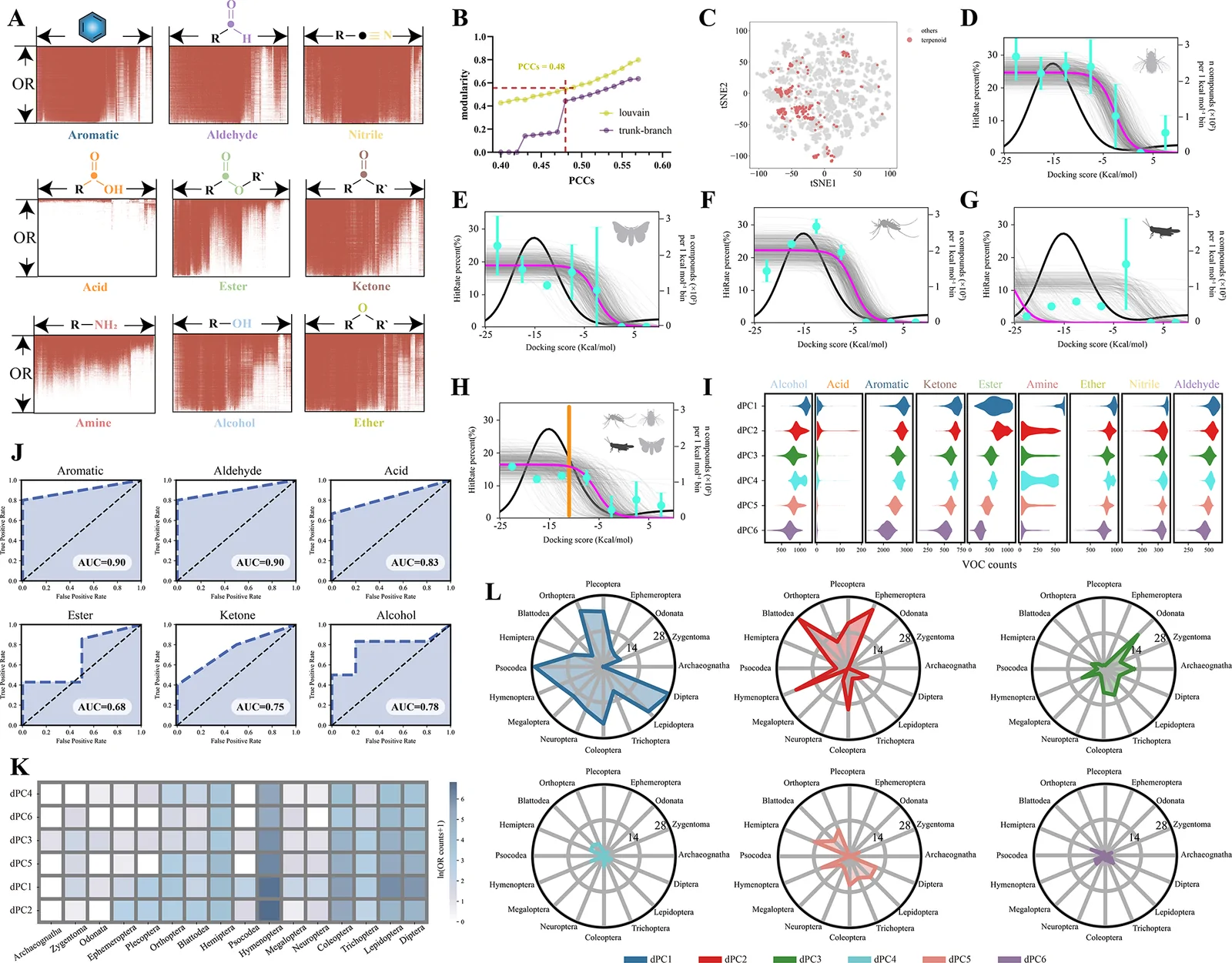
Insect OR–VOC binding profiles, dPC labeling accuracy, and distribution of docking-profile communities across insect orders. (**A**) Binding profiles of insect ORs with VOCs from different functional groups. Each point represents the interaction between an OR and a small molecule. A docking score below −8 kcal/mol was classified as predicted binding. (**B**) Modularity changes between docking-profile communities at different PCC thresholds. Purple represents the docking-profile community modularity obtained using the ‘trunk–branch’ algorithm, while yellow denotes the theoretical optimal community modularity based on the Louvain algorithm. (**C**) Distribution of terpenoids in the two-dimensional odor space. (**D–H**) Hit rates for ORs from single insect taxa. Species include *Drosophila melanogaster, Anopheles gambiae, Helicoverpa armigera, Spodoptera littoralis*, and *Locusta migratoria*. Cyan dots represent the mean hit rate ± SEM for each docking-score interval. The gray curve represents posterior distributions from Bayesian inference, with n=500. The black curve shows the distribution of hit rates and docking scores across all OR–VOC docking results. (**I**) Number of VOCs recognized by different dPCs. Colors represent different dPCs, consistent with Figure 5C. (**J**) Accuracy of dPC labeling using *Drosophila* ORs as an example. The AUC represents the relationship between the predicted small molecule recognition tendency labels and the experimentally validated number of recognized VOCs. (**K**) Number of ORs in different docking-profile communities across insect orders. Due to large variations in OR numbers among orders, the OR counts are ln-transformed. (**L**) Wilson scores for different dPCs across insect orders. Wilson scoring was applied to correct for potential errors caused by low OR numbers in certain orders, providing a more balanced evaluation of dPC representation.

**Figure 5—figure supplement 2.**
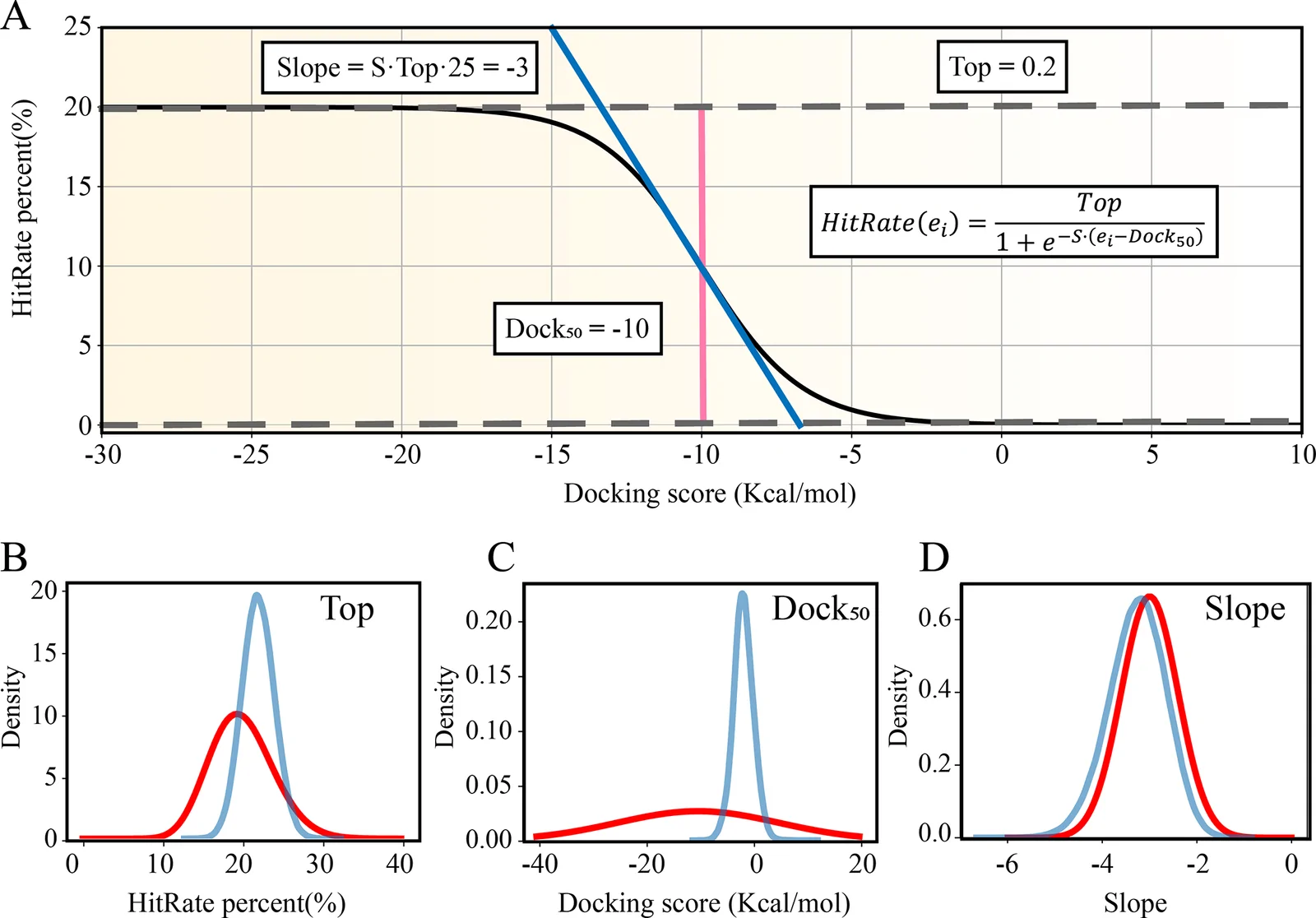
Bayesian prior modeling to optimize VOC selection by balancing information acquisition and ligand discovery. (**A**) Sigmoid functional form for the hit-rate model. (**B–D**) Marginal Bayesian prior (blue) and posterior (red) distributions (n=100,000) for each model parameter. (**B**) Top. (**C**) Dock50. (**D**) Slope.

We performed molecular docking for all insect ORs using these VOCs to generate their docking-derived binding profiles (Figure 5—figure supplement 1A). A long-standing challenge in molecular docking is how to predict binding likelihood on the basis of ranking scores. To address this issue, we modeled a theoretical ‘hit rate’ curve using docking scores and experimental datasets from *Drosophila* (Hallem and Carlson, 2006), mosquitoes (Carey et al., 2010), and moths (de Fouchier et al., 2017; Guo et al., 2021; Figure 5B). Using a Bayesian framework with prior probability distributions, we determined an informative docking-score range for insect ORs. A threshold score of –8 kcal/mol was established to maximize data retention while maintaining a reliable hit rate (Figure 5B). In addition, our single-taxon hit-rate sensitivity analysis showed similar trends for *Drosophila*, *Anopheles*, and moths (Figure 5—figure supplement 1D–F). Because the locust OR repertoire had an extremely low positive rate, we did not include the locust experimental dataset in the final threshold assessment (Figure 5—figure supplement 1G and H, see Appendix 2 for details). Although this threshold enriches experimentally responsive OR–VOC pairs, the resulting hit rate is not sufficient to support deterministic inference for individual receptor-ligand pairs. Therefore, subsequent analyses were interpreted at the OR repertoire level as docking-derived comparative patterns rather than as experimentally validated OR response spectra.

We constructed a dPSN for ORs (Figure 5C). In this network, each node represents ORs with a docking-profile Pearson correlation coefficient (PCC) greater than 0.6, and nodes with an average PCC greater than 0.48 are connected by edges. We identified six docking-profile communities (dPCs), representing 72.5% of the ORs (Figure 5—figure supplement 1B). It should be noted that dPCs represent docking-profile communities rather than experimentally confirmed functional classes. We subsequently quantified the number of VOCs from different functional groups meeting the docking threshold for each dPC (Figure 5—figure supplement 1I) and assigned docking-derived binding labels on the basis of their relative enrichment compared with the background (Figure 5D). These labels summarize the predicted breadth of VOC functional-group binding for each dPC. To evaluate the extent to which these docking-derived labels were consistent with available experimental data, we calculated the area under the receiver operating characteristic curve (AUC) by comparing experimental OR response data with the corresponding docking-derived labels. For most functional group predictions, an AUC exceeding 0.75 was achieved, suggesting that these labels capture broad directional tendencies in available experimental data (Figure 5—figure supplement 1J). dPC1 showed a relatively broad docking profile across VOC functional groups, whereas dPC6 showed a narrower docking profile (Figure 5D). Additionally, dPC3 showed relatively stronger predicted binding toward aromatics, whereas dPC5 showed relatively stronger predicted binding toward nitriles.

Unlike SeqC and StrC, almost all insect orders contain all dPCs without exhibiting specificity (Figure 5—figure supplement 1K). dPC1, characterized by a relatively broad docking profile, accounted for the largest proportion of ORs in most orders, particularly Psocodea, Diptera, and Lepidoptera (Figure 5—figure supplement 1L). In contrast, dPC6, characterized by a relatively narrow docking profile, represented only a small proportion of ORs across the sampled species.

### Comparative patterns of insect OR diversification across evolutionary periods

**Figure 6.**
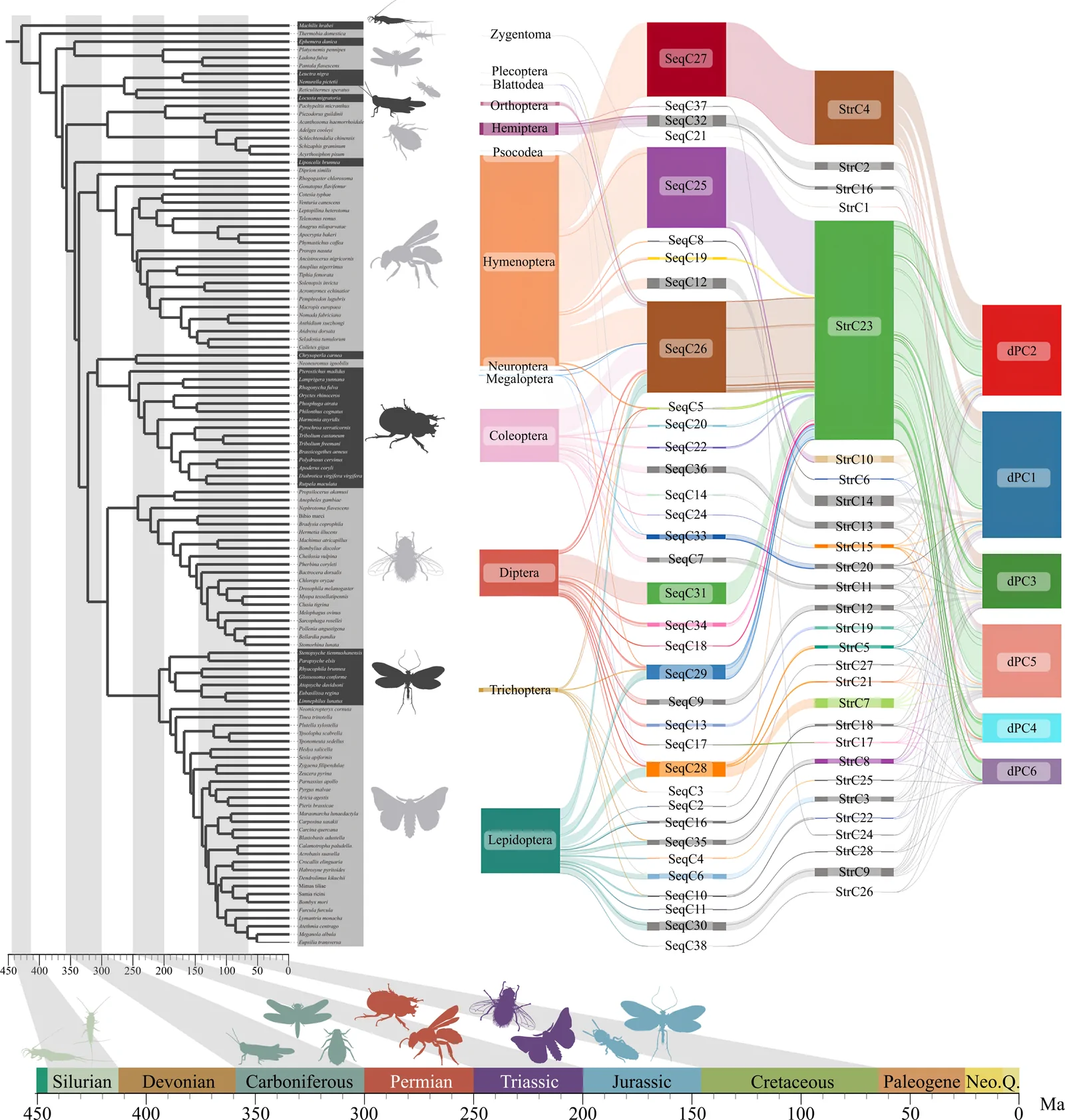
Relationships between OR sequence, structure, and docking-profile communities across insect orders (Figure 6—source data 1). The phylogenetic tree follows the structure from Figure 1. On the right, the mapping relationships between all insect OR SeqCs, StrCs, and dPCs are shown, considering only ORs classified into communities. Time nodes for different geological periods are based on the previous study (Misof et al., 2014) and are separated by gray shading. The timeline beneath the tree marks the origin of various insect orders. Animal silhouettes were obtained from PhyloPic.org. Figure 6—source data 1.Community annotation of insect ORs.

Although the sequence evolution of the insect OR gene family and functional diversity among different orders and species have been reported multiple times (Brand and Ramírez, 2017; McKenzie and Kronauer, 2018; Legan et al., 2021; Chang et al., 2023), these studies reveal only a partial view rather than the broader patterns of insect OR sequence, structural, and docking-profile diversity. To characterize large-scale diversification patterns of insect ORs, we integrated SeqCs, StrCs, and dPCs. The results revealed strong congruence between SeqCs and StrCs, with both communities including nearly all dPCs (Figure 6, Figure 6—figure supplement 1). This pattern suggests that sequence and structural diversification are more closely coupled, whereas docking-profile variation shows a weaker correspondence with either level.

**Figure 6—figure supplement 1.**
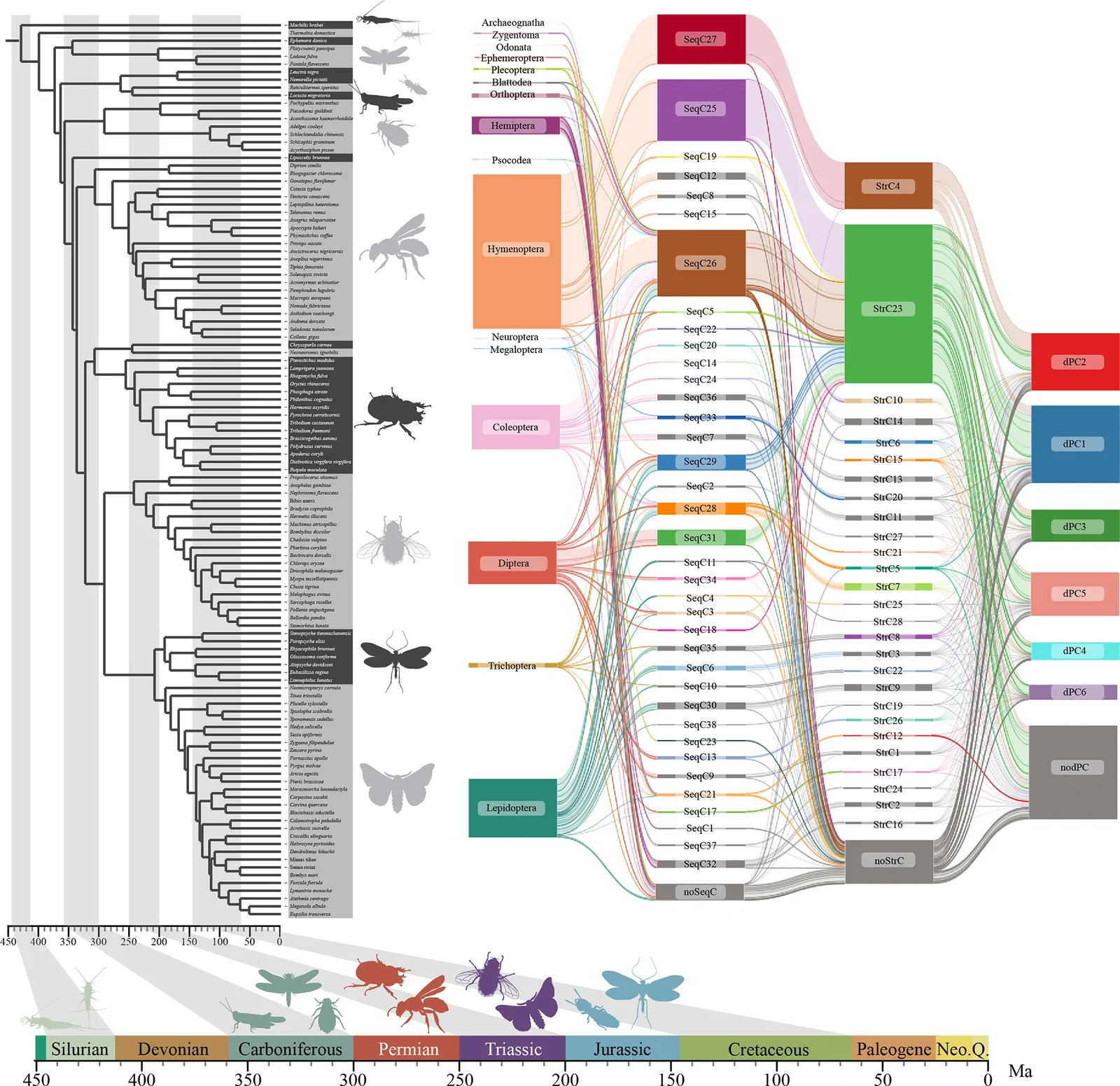
Relationships between OR sequence, structure, and docking-profile communities across insect orders (all ORs). The phylogenetic tree follows the structure from Figure 1. On the right, the mapping relationships between all insect OR SeqCs, StrCs, and dPCs are shown. Time nodes for different geological periods are based on previous studies (Misof et al., 2014) and are separated by gray shading. The timeline beneath the tree marks the origin of various insect orders. Animal silhouettes were obtained from PhyloPic.org.

**Figure 6—figure supplement 2.**
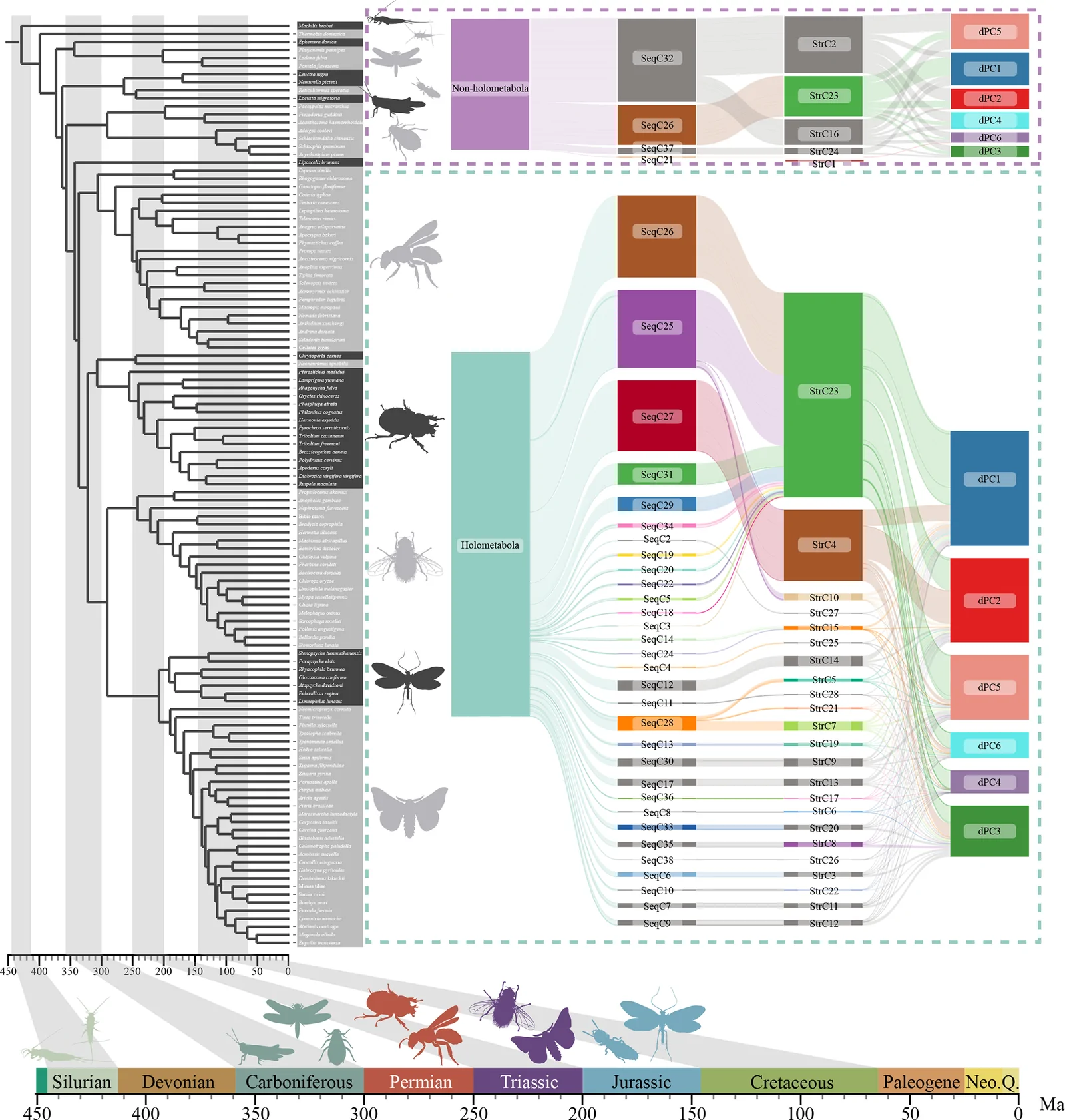
Sequence, structural, and docking-profile community associations of ORs in holometabolous and non-holometabolous insects. The phylogenetic tree follows the structure from Figure 1. On the right, the mapping relationships between all insect OR SeqCs, StrCs, and dPCs are shown, considering only ORs classified into specific communities. Time nodes for different geological periods are based on previous studies (Misof et al., 2014) and are separated by gray shading. The timeline beneath the tree marks the origin of various insect orders. Animal silhouettes were obtained from PhyloPic.org.

**Figure 6—figure supplement 3.**
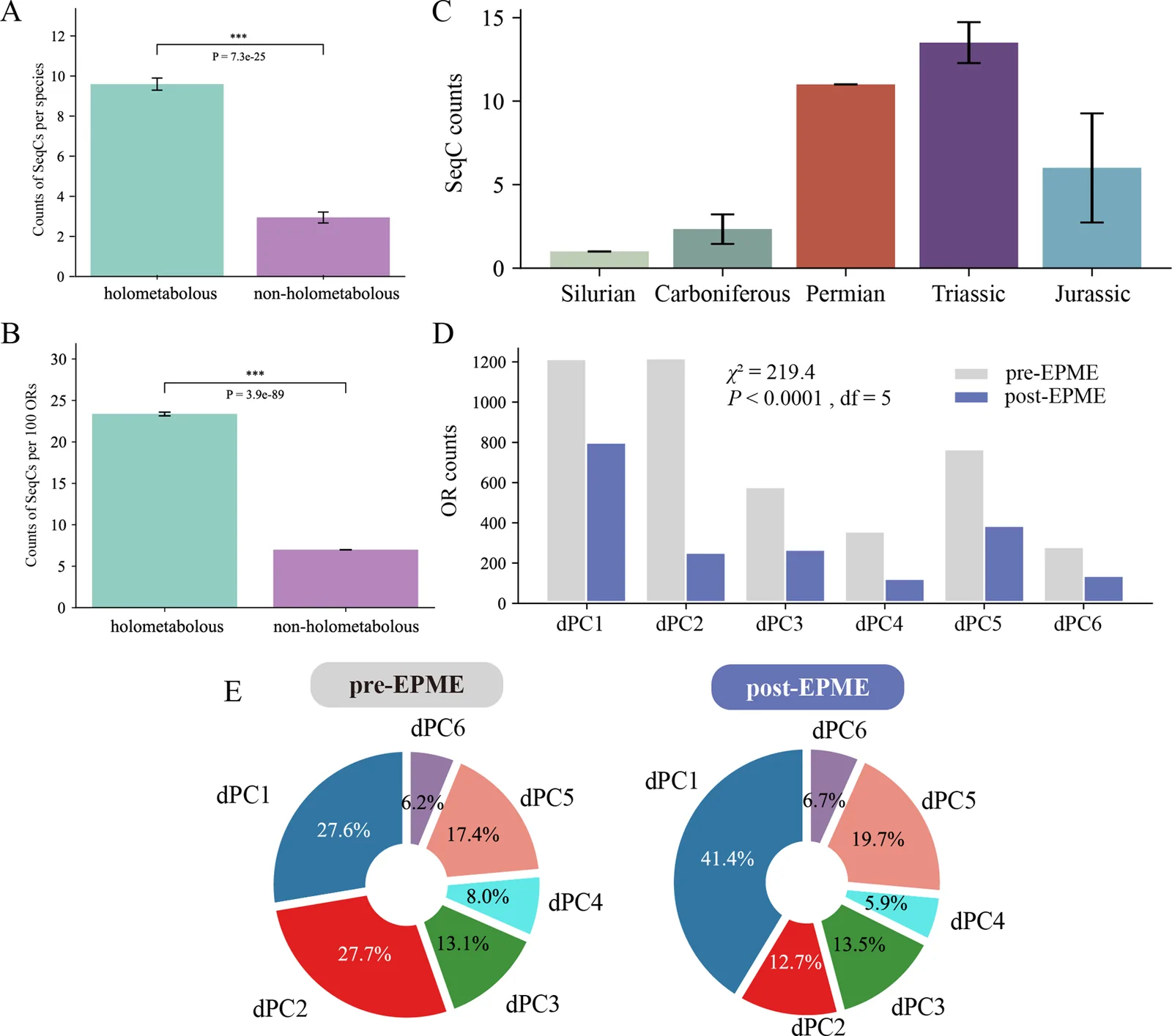
Changes in insect OR SeqC types and dPC proportions across different geological periods. (**A–B**) Normalized analysis of SeqC diversity in holometabolous and non-holometabolous insects at the species level (**A**) and OR-count level (**B**) Error bars represent means ± SEM; n = 97 holometabolous species and 18 non-holometabolous species. Welch’s t-test was used. ***p<0.001. (**C**) The number of SeqC types in insect ORs across different geological periods. (**D**) Changes in the number of ORs within different dPC categories before and after the EPME. (**E**) Proportional changes in different dPCs of insect ORs before and after the EPME. EPME, end-Permian mass extinction.

**Figure 6—figure supplement 4.**
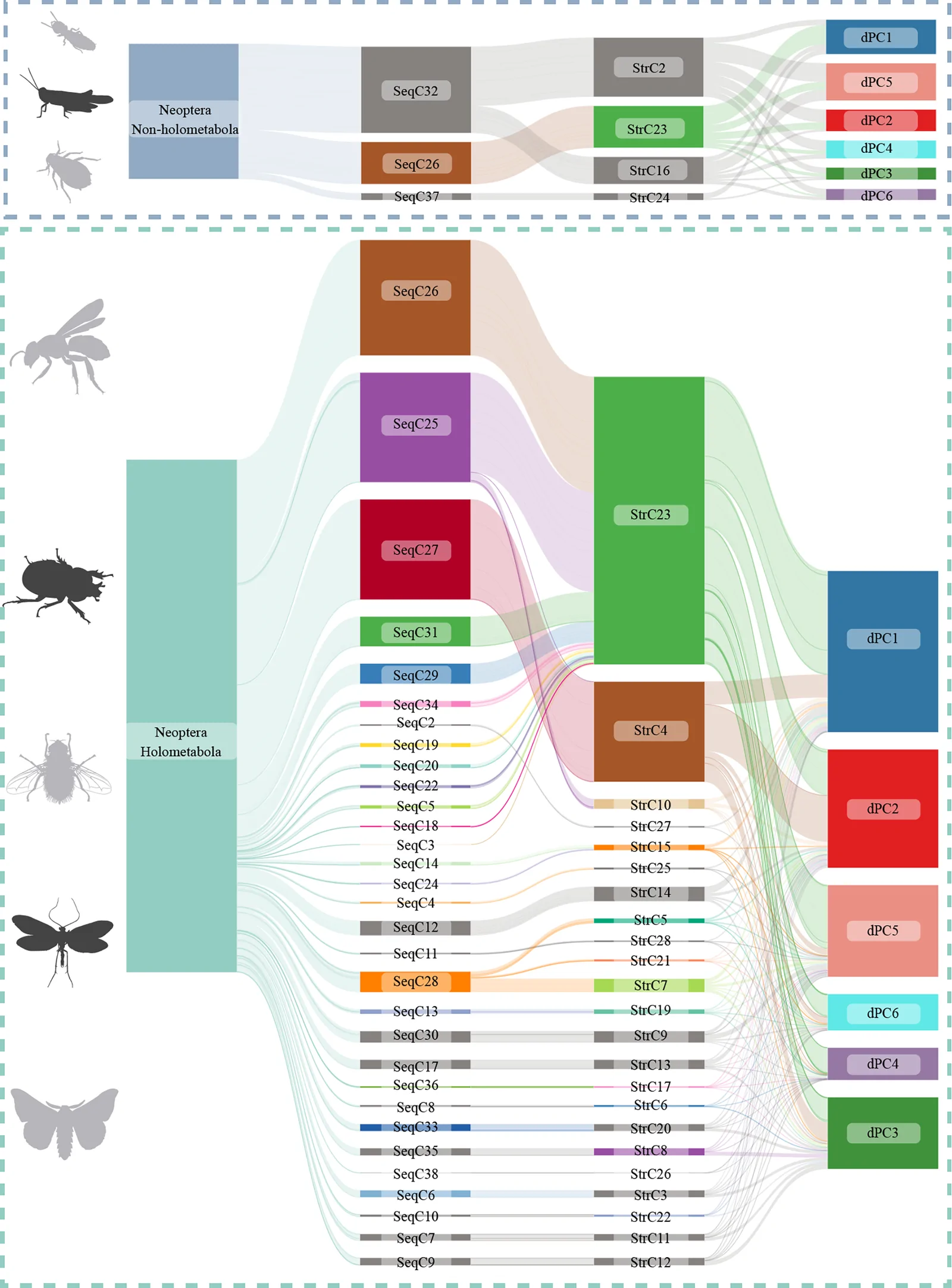
Comparison of OR SeqC diversity between Holometabola and non-holometabolous Neoptera.

Additionally, extant holometabolous insects showed higher OR sequence-community diversity than non-holometabolous insects (Figure 6—figure supplement 2). Because total SeqC counts may be affected by unequal species sampling and OR numbers, we added normalized analyses. First, we calculated SeqC numbers at the species level and found that holometabolous insects contained more SeqCs on average (Figure 6—figure supplement 3A). Second, because OR number was significantly correlated with SeqC count (p=0.001035), we performed equal-OR random sampling between holometabolous and non-holometabolous insects. Under the same OR sampling depth, holometabolous insects still covered more SeqCs, indicating higher OR sequence-community diversity after controlling for OR number (Figure 6—figure supplement 3B). Therefore, this result supports a genuine difference in SeqC diversity rather than a difference driven by unequal species sampling or OR number. The higher SeqC diversity observed in Holometabola was retained when the comparison was restricted to Neoptera, indicating that this pattern is not simply a restatement of previously reported Paleoptera-Neoptera differences (Figure 6—figure supplement 4).

The number of SeqCs increased with deeper divergence among insect orders, suggesting gradual accumulation of OR sequence variation during insect evolution (Figure 6—figure supplement 3C). Despite the limited SeqC diversity in non-holometabolous insects, all six dPC types were represented in these extant lineages, suggesting that the major docking-profile community types may have arisen relatively early in insect evolution.

The EPME was a significant geological event that resulted in the extinction of most species and caused drastic environmental changes (Close et al., 2020; Jouault et al., 2022). In the extant species sampled here, we detected differences in dPC composition between lineages whose order-level origins fall before and after the EPME (Figure 6—figure supplement 3D). Lineages originating after the EPME showed a greater proportion of dPC1, which was characterized by a broader docking profile, and a lower proportion of dPC2 (Figure 6—figure supplement 3E). This pattern is consistent with previously reported data that the proportion of broad-tuned ORs in *Locusta* is much smaller than that in *Drosophila* and *Anopheles* (Chang et al., 2023). These observations should be interpreted as a docking-derived comparative pattern among extant lineages rather than direct evidence of ancestral functional changes across the EPME.

## Discussion

In this study, we integrated sequence, structural, and docking-profile analyses to characterize large-scale patterns of insect OR diversification. Across the sampled insect species, OR repertoire size was associated with several ecological traits. Sequence and structural communities showed a close correspondence, whereas docking-profile communities were less closely associated with either classification. Differences in dPC composition among extant lineages whose order-level origins predate or postdate the EPME raise the hypothesis that these differences may be associated with ecological changes surrounding this event. Within the ‘conserved chassis-diversified sensors’ model, we further examined Orco specialization and identified the conserved EL2 β-sheet and distinctive binding-pocket properties as structural features that may contribute to reduced odorant-binding capacity.

The long-IL3 OR orthologous group includes *Drosophila melanogaster* DmOr13a and *Bactrocera dorsalis* BdorOR13a, both of which respond to 1-octen-3-ol and belong to a group of dipteran fly OR homologs associated with oviposition behavior (Kreher et al., 2008; Liu et al., 2023; Xu et al., 2023). Modeling of these homologous ORs showed that they also contain a long IL3 region. We therefore propose that long-IL3 ORs correspond to these fly homologs that recognize 1-octen-3-ol. However, 1-octen-3-ol has different behavioral functions in different species: it acts as an attractant in blood-feeding mosquitoes and tsetse flies (Hall et al., 1984; Kline et al., 2007), whereas in flies it is usually associated with oviposition regulation (Kreher et al., 2008; Liu et al., 2023). Mosquito receptors known to detect 1-octen-3-ol, such as AgOR8, AaOR8, TaOR8, and CquiOR118b (Lu et al., 2007; Xu et al., 2015; Dekel et al., 2016; Frunze et al., 2024), do not contain a long IL3 region (Figure 3—figure supplement 1E). This suggests that the long IL3 structure in fly homologs may be related to the oviposition-associated role of 1-octen-3-ol in flies, which requires future experimental validation.

Despite substantial differences in OR sequence and structural community composition, nearly all sampled insect orders contained representatives of all major docking-profile communities. SeqCs and StrCs showed a closer correspondence with each other than either did with dPCs, suggesting that sequence and structural diversification are more tightly coupled, whereas variation in docking profiles is less directly associated with either level. Thus, distinct sequence and structural repertoires can converge on broadly similar distributions of docking-profile communities across insect orders. This pattern suggests that diversification in sequence and structure does not necessarily translate into equivalent divergence in predicted ligand-binding profiles. We also detected differences in dPC composition between extant lineages whose order-level origins predate or postdate the EPME. In particular, lineages whose order-level origins postdate the EPME had a higher proportion of dPC1, a community characterized by a relatively broad docking profile. The Permian–Triassic transition was accompanied by extensive ecological disruption and major restructuring of terrestrial ecosystems, including substantial changes in plant communities and insect–plant interactions (Grauvogel-Stamm and Ash, 2005; Benton, 2016; Vajda et al., 2020; Lu et al., 2021; Jouault et al., 2022). These environmental changes raise the hypothesis that ecological turnover surrounding the EPME may have contributed to differences in OR docking-profile composition among insect lineages. However, this interpretation remains hypothetical. Because rapid OR evolution and limited cross-order orthology preclude reliable reconstruction of deep ancestral repertoires, the EPME-related pattern should be viewed as a descriptive comparison among extant lineages. Further validation will require broader taxon sampling, ancestral reconstruction, and experimental testing of receptor tuning breadth.

Previous studies have proposed an evolutionary transition from ancestral chemoreceptors to the insect OR–Orco system, and our results provide structural insight into the specialization of Orco within this framework (Brand et al., 2018; Thoma et al., 2019). The ancestral insect recruited GRs to perform olfactory functions, giving rise to the earliest ORs (Robertson et al., 2003; Frank et al., 2024; Mitchell et al., 2026). These early ORs retained the homotetrameric architecture of GRs (Del Mármol et al., 2021). However, the sensitivity requirements of olfaction and gustation differ (de Fouchier et al., 2017; Pask et al., 2017; Slone et al., 2017; Dweck and Carlson, 2023; Wang et al., 2024; Zhao et al., 2024). This issue appears to have been first addressed in the ancestor of Zygentoma and Pterygota by reducing the number of functional subunits while maintaining the tetrameric structure, thereby increasing sensitivity (Brand et al., 2018; Thoma et al., 2019; Wang et al., 2024; Zhao et al., 2024). Two structural features are consistently conserved among Orcos: the EL2 β-sheet and the highly specialized binding pocket. The β-sheet physically prevents VOCs from accessing the binding pocket, whereas the chemically restrictive nature of the binding pocket prevents VOCs from penetrating deeply. Orco exhibits strong sequence conservation, ensuring that these chemical and physical barriers are effectively preserved. Sequence similarity is much lower in the insect OR family than in the GR family, which may be related to the emergence of Orco. This division between a conserved co-receptor and diversified ligand-selective subunits has been proposed to provide a stable structural framework within which OR diversification can occur (Zhao et al., 2024). However, we also identified several ligand-selective ORs containing an Orco-like EL2 β-sheet. Whether this structural similarity reflects shared ancestral features, convergent structural evolution, or other evolutionary processes remains unresolved (Figure 4—figure supplement 1E and F).

Overall, we characterized the relationships among sequence, structure, and docking profiles in insect ORs, identified structural features potentially associated with Orco specialization, and detected class-wide associations between OR repertoire size and ecological traits. The resulting dataset and similarity-based community framework provide a resource for insect OR research, while the docking-profile patterns offer testable hypotheses for future experimental and evolutionary studies.

## Materials and methods

### Genome data

We compiled data from all published insect genomes available in the NCBI public database as of December 2022. Genomes with a contig N50>1 Mb were considered reliable for OR annotation. When multiple high-quality genomes were available within a single insect family, we selected the genome with the highest assembly quality as the representative. After filtering, 107 insect genomes were included: Odonata (2), Plecoptera (2), Blattodea (1), Hemiptera (6), Psocodea (1), Hymenoptera (22), Megaloptera (1), Neuroptera (1), Coleoptera (14), Trichoptera (7), Lepidoptera (30), and Diptera (20). Detailed genome information is provided in the Figure 1—source data 1. To maximize phylogenetic diversity, we added eight representative species from different orders with annotated ORs: *M. hrabei* (Brand et al., 2018), *T. domestica* (Brand et al., 2018), *Ladona fulva* (Brand et al., 2018), *Ephemera danica* (Brand et al., 2018), *Locusta migratoria* (Wang et al., 2015), *Acyrthosiphon pisum* (Robertson et al., 2019), *Acromyrmex echinatior* (Zhou et al., 2015), and *Tribolium castaneum* (RRID:SCR_010440) (Engsontia et al., 2008). In total, our study includes 115 insect species, covering 16 orders and 111 families.

### Species phylogenies

To construct the phylogenetic tree used in this study, we merged existing phylogenies across different insect orders (Misof et al., 2014). For relationships among orders within Insecta, we referred to previously published phylogenetic frameworks. To ensure broad taxonomic representation, we included 16 insect orders in our analysis. Intra-order relationships were obtained from insectphylo.org and other published species trees covering various insect taxa (Chesters, 2017). For species with available genomes that were not included in existing phylogenies, we inferred their phylogenetic placements based on family-level information. Divergence times among orders were obtained from TimeTree.org (Kumar et al., 2022) and calibrated using MCMCtree (Rannala and Yang, 2007). The resulting phylogeny includes 115 insect species spanning 16 orders.

### Ecological trait data of insect species

Given the vast diversity and ecological complexity of insects, and the lack of a comprehensive authoritative database, we manually retrieved ecological trait data from published literature for each species. We characterized insect ecology based on four aspects: larval diet, adult diet, habitat, and circadian rhythm. For dietary traits, we classified diets into 12 types: herbivorous, xylophagous, hematophagy, mycophagous, non-feeding, omnivorous, predatory, saprovorous, frugivorous, nectarivorous, detritivorous, and parasitic. For habitat, following previous classification criteria, species were assigned as either terrestrial or aquatic (Dijkstra et al., 2014). Insects that live entirely or have at least one life stage in water were considered aquatic; those that are strictly land-dwelling throughout their life cycle were considered terrestrial. Circadian rhythm was classified based on adult activity periods as either diurnal or nocturnal. The ecological classification and corresponding literature sources for each species are provided in the Figure 1—source data 2.

### OR gene mining and structural filtering

All OR and Orco protein sequences were downloaded from NCBI, supplemented with ORs reported in the paper, yielding a total of 16,176 ORs. Redundancy and data cleaning were then performed using CD-HIT (Li and Godzik, 2006; Fu et al., 2012) v4.8.1 to remove highly similar sequences with >90% identity, resulting in 6029 non-redundant ORs used as queries. We used Exonerate (Slater and Birney, 2005) v2.4.0 to search whole-genome data and identify potential exon regions. To improve annotation efficiency, genome contigs were split into 20 Mb segments, with 20 kb flanks added at breakpoints to avoid truncating ORs. Next, InsectOR (Karpe et al., 2021) was used for OR gene annotation.

Given that multi-exon gene annotation from genome can be error-prone, we applied the strictest structural filtering strategy to enhance annotation reliability (Figure 1—figure supplement 1A). All annotated ORs were modeled using AlphaFold2 (Jumper et al., 2021). Structural alignment against the cryo-EM structure of MhOR5 (PDB: 7LIC) was performed using Dali (Holm, 2022), and ORs with Z-scores <20 were excluded. We further manually removed sequences lacking at least seven transmembrane domains. In total, 8905 ORs with complete and credible structural models were retained for downstream analyses. This rigorous approach eliminates structurally compromised ORs that may appear intact at the sequence level, thereby increasing the likelihood of identifying ORs with functional physiological and biochemical properties.

### Phylogenetic comparative analyses

The function pGLS of the R package caper was used to perform all phylogenetic generalized least squares models presented in this study. Phylogenetic manipulations were conducted using the R packages phytools (RRID:SCR_015502) and ape. Phylogenetic trees were visualized by iTOL (Letunic and Bork, 2024; https://itol.embl.de/). Animal silhouettes used in this study were retrieved from http://phylopic.org/.

### ‘Trunk–Branch’ strategy

Most existing insect ORs have arisen through gene duplication events (McKenzie and Kronauer, 2018; Legan et al., 2021). Initially, these duplicated OR sequences were highly similar, forming the ‘trunk’ of core communities. Over time, mutations led ORs to diverge from their original sequences and functional features, forming the ‘branches’ of the community (Figure 2A). Based on this ‘trunk–branch’ concept, we developed a strategy to construct similarity networks and define communities and the specific implementation process is as follows:

1. Communities with more than 20 nodes in the initial-threshold network are defined as core communities (trunks). The initial thresholds were set at e-value=1e-68 for the SSN and Z-score=45.9 for the StSN.
2. Thresholds were gradually relaxed to extend the branches of each core community. Newly appearing nodes were assigned to the community with which they shared the highest number of edges. The SSN threshold was relaxed in logarithmic steps (e.g. 1e-68, 1e-67,...), while the StSN threshold was relaxed in steps of 0.1 Z-score (e.g. 45.9, 45.8,...).
3. Communities were merged based on their inter-community connectivity. If the proportion of edges connecting two communities exceeded 50% for either community, they were merged. This process was repeated until all inter-community connectivities fell below the 50% threshold.
4. New communities with more than 20 unassigned nodes were identified and included.
5. Steps 2–4 were repeated until the network reached a stage of edge explosion and massive community merging.

Previously, final visualization thresholds for sequence similarity networks were often chosen based on known functional annotations. However, such information is lacking for most ORs. We found that under different thresholds, the modularity of community structures defined by the ‘trunk–branch’ strategy showed a sharp inflection point when compared with theoretical modularity derived from the Louvain algorithm. Here, we propose a quantifiable approach for determining the final community display threshold by identifying the inflection point. Validation against known data confirmed that the resulting community partitions are highly reliable.

### Sequence similarity network

First, we performed pairwise sequence alignment of all ORs using MMseqs2 (Steinegger and Söding, 2017) to obtain sequence similarities and e-values. To improve network readability, we applied pre-clustering with CD-HIT (Li and Godzik, 2006; Fu et al., 2012) v4.8.1 (‘-c 0.6 n 4’) to reduce redundant edges between highly similar nodes, resulting in a total of 4947 nodes. The edge weight between nodes was defined as the average e-value of all OR pairs they represent. We then constructed the sequence similarity network based on the ‘trunk–branch’ strategy (see ‘Trunk–branch strategy’). An e-value threshold of 1e-25 was ultimately selected as the cutoff for defining the final sequence similarity network and OR subfamilies. At this threshold, 37 OR communities were identified, comprising 8,457 ORs. The network was visualized using the organic layout algorithm in Cytoscape (Shannon et al., 2003).

### Structure similarity network

We performed structural modeling of all ORs using AlphaFold2 (Jumper et al., 2021). Pairwise structural comparisons were then conducted using Dali (Holm, 2022) to obtain Z-scores. Following an approach similar to that used in SSN, we applied a greedy clustering algorithm to pre-cluster structurally similar ORs at a Z-score threshold of 50, resulting in 3854 nodes. The edge weight between nodes was defined as the average Z-score of all OR pairs they represent. We then constructed the structure similarity network using the ‘trunk–branch’ strategy. A Z-score threshold of 42.9 was selected as the final cutoff for defining the StSN and OR structural subfamilies. At this threshold, 27 OR structural communities were identified, comprising 7750 ORs. The network was visualized using the organic layout algorithm in Cytoscape (Shannon et al., 2003).

### Phylogenetic tree and selection pressure analysis

To infer the amino acid phylogeny of ORs within StrC17, we performed multiple sequence alignment using MAFFT (Katoh and Standley, 2013) v7.505 with the parameters ‘--globalpair --maxiterate 1000’. The alignments were trimmed using trimAl (Capella-Gutiérrez et al., 2009) v1.4.rev22 with the parameter ‘-automated1’. A maximum likelihood tree was then constructed using IQ-TREE (Minh et al., 2013; Nguyen et al., 2015) v 2.2.0 with the parameters ‘-m MFP -B 1000 --bnni’. To estimate dN/dS ratios, we used the codeml program from the PAML package (RRID:SCR_014932; Yang, 1997; Yang, 2007). The codeml analysis employed the branch model with the control file parameters set to ‘model = 2’, ‘NSsites = 0’. We used a custom script to extract log-likelihood values (lnL) from the output files and perform likelihood ratio tests (LRTs).

### Evaluation of OR binding pocket properties

We used fpocket to identify OR binding pockets and assess their properties, with parameters set to ‘-m 3.1 -i 10’. To locate the centroid region of the binding pocket, we aligned each OR structure to MhOR5 using USalign (Zhang et al., 2022) v 20220924. Among all candidate pockets identified by fpocket (Le Guilloux et al., 2009) 4.0, the one closest to the centroid was designated as the binding pocket. Due to the stochastic nature of OR modeling, some ORs exhibited open binding pockets, making it difficult to define cavity-related parameters. To ensure consistency in the quantitative analysis, we only included ORs with closed binding pockets. Specifically, we retained only binding pockets with Number of Alpha Spheres <140 and Volume <800, excluding any that were open to the exterior.

### Molecular dynamics simulation of Orco β-sheet structural function

We used GROMACS (Abraham et al., 2015) to simulate the interaction between the EL2 regions of OR and Orco with VOCs. The proteins used were the cryo-EM structures of ApOrco and ApOR5 (Wang et al., 2024; PDB: 8z9z), and the selected VOC was geranyl acetate (CID: 1549026). The membrane system was rapidly constructed using the QwikMD plugin in VMD (Humphrey et al., 1996). After default minimization and annealing steps, an optimized membrane-protein system was obtained. The Caver (Jurcik et al., 2018) tool was used to identify the pocket tunnels in the cryo-EM structures of ApisOR5 and ApisOrco. Surface residues were identified using FindSurfaceResidues (https://pymolwiki.org/index.php/FindSurfaceResidues), helping define the residues surrounding the binding pocket. Geranyl acetate was positioned above the pocket, and 5 ns MD simulations were performed to evaluate interactions.

A binding state was considered potential if the following criteria were met:

- The minimum distance between geranyl acetate atoms and pocket residue atoms was <12 Å (cutoff).
- The spatial distribution of the ligand satisfied the following exclusion conditions:
- Not located on the lateral side of the protein
- Not embedded in the phospholipid bilayer
- Not sterically blocked by the EL2 of ApisOrco

Each simulation was repeated 60 times for both ApOrco and ApOR5.

It should be noted that the MD simulations were designed to examine the early trajectories of VOCs near the extracellular entrance of the binding pocket, rather than to assess the conformational stability of the receptor complex. A duration of 5 ns is sufficient for small molecules to respond to local steric constraints.

### Cell lines

HEK293T cells (RRID:CVCL_0063) were purchased from Wuhan Procell Biotechnology, China. Cell line identity was authenticated by STR profiling performed by Wuhan Procell. Cells were tested for mycoplasma contamination every three months and were negative. Cells were maintained in DMEM (BasalMedia, China) supplemented with 10% fetal bovine serum (ExPhos, China) and 1% penicillin–streptomycin (Sangon Biotech, China) at 37 °C in a humidified atmosphere containing 5% CO_2_.

### Heterologous expression and calcium imaging

pDmelOR-mRho.V5.mER.hOr47a (RRID:Addgene_126478), a high-copy number bidirectional expression vector by inserting *D. melanogaster* Or47a (DmOr47a) and Orco in the pCMV-BI vector (RRID:Addgene_126475) backbone (Miazzi et al., 2019), was a gift from Bill Hansson (Addgene plasmid # 126478), then the V5 tag was replaced with the Flag tag, named pDmelOR-OR47a. The EL2 of OR47a (GHAEPELPFPCLFPWNIHI) in pDmelOR-OR47a was replaced by the EL2 of Orco (GDSVKMVVDHETNSSIPVEIPRLPIKSFYPWNASHG) to construct the new plasmid of pDmelOR-el2.OR47a. The sequences were confirmed by Sanger sequencing (Sangon Biotech, China).

At 70–80% confluency, HEK293T cells were transfected with plasmids using LipoFiter transfection reagent (Hanbio, China). At 6–8 hr after transfection, cells were seeded at a density of 6×10^5^ cells per well into 35 mm glass-bottom culture dishes (NEST, China). Cells were harvested for calcium imaging approximately 36 hr post-transfection. The culture medium was removed, and cells were rinsed with HBSS solution (BasalMedia, China). Subsequently, cells were incubated with Fluo-8 AM (5 μM, AAT Bioquest, USA) diluted in HBSS + 0.04% pluronic acid (F127, Sangon Biotech, China) in a CO_2_ cell culture incubator for 30 min at 37 °C. Following three washes with HBSS solution, the Ca^2+^-dependent fluorescence was recorded with a fluorescence microscope (DeltaVision Ultra, GE) with the emission wavelength set at 525 nm and the excitation wavelength at 490 nm. Experiments lasted 5 min with a sampling interval of 5 s. The stimuli—PA (100  μM; Aladdin, China), an OR47a agonist, and VUAA1 (100  μM; Sigma-Aldrich, USA), an Orco agonist—were gently added to the culture dish at 50  s and 180  s, respectively, after the start of the imaging protocol. The recorded signals were analyzed by Fiji (RRID:SCR_002285) ImageJ v.1.54 (RRID:SCR_003070) (Schindelin et al., 2012). Levels of Ca^2+^ were expressed as ΔF/F0 ratios. The average fluorescence intensity of the cells during the first 30 seconds was recorded as the baseline (F0). Each assay was conducted in triplicate to ensure reproducibility. For calcium imaging experiments, data acquisition and initial ΔF/F0 ratio calculations were performed by researchers who were blinded to the treatment groups during the analysis phase. Sample sizes for calcium imaging (n=30) were determined based on common standards in the field and prior pilot experiments to ensure sufficient statistical power for detecting changes in fluorescence intensity.

### Virtual docking data of ORs and VOCs

We constructed an insect-relevant odorant molecule library from four sources: pheromones (Li et al., 2023), food odors (Chi et al., 2025), fragrances (thegoodscentscompany.com), and theoretical odors (Mayhew et al., 2022). This approach ensured ecological relevance and chemical diversity. After deduplication, a total of 14,499 VOCs were retained for downstream analysis. We used Dragon (v.5.4)(Haddad et al., 2008) to calculate 32 representative molecular descriptors for each VOC, and the ChemmineR (Cao et al., 2008) R package to identify functional groups. VOCs were assigned functional group labels based on group priority rules. Dimensionality reduction of the 32 descriptors was performed using t-SNE, and the resulting two-dimensional odor space was visualized using Matplotlib (Hunter, 2007). All VOC SMILES were converted to db2 format using the online resource tldr.docking.org. OR structures were aligned to the cryo-EM structure of MhOR5 (PDB:7LIC) to estimate the approximate position of the binding pocket. Virtual docking was then performed using DOCK 3.8 (RRID:SCR_000128; Coleman et al., 2013), with batch processing handled via custom scripts ‘prepare_dock_file.sh’ and ‘run_dock_paraFly.sh’. In total, we obtained 130 million docking records between ORs and VOCs.

### Hit rate curve prediction and maximum hit estimation

We first compiled published functional datasets containing explicit OR–VOC response relationships. These datasets included ORs from *D. melanogaster*, *Anopheles gambiae*, *Helicoverpa armigera*, *Spodoptera littoralis,* and *L. migratoria*. For each dataset, OR–VOC combinations were assigned response or non-response labels according to the original experimental results. Each OR–VOC pair was then docked using the same structural modeling, docking, and scoring workflow used in the global OR–VOC analysis. Pairs were grouped into docking-score intervals, and the hit rate for each interval was defined as the proportion of experimentally positive pairs among all pairs in that interval. Each score interval was sampled three times. To determine the docking score range at which VOCs are likely to exhibit binding activity in electrophysiological assays, we applied Bayesian statistical modeling using prior probability distributions to define the top, midpoint, random hit rate, and slope of the hit rate curve. We assumed that the relationship between docking score (energy, ei) and hit rate follows a dose-response-like curve (Figure 5—figure supplement 2A; Lyu et al., 2019).

$$\displaystyle HitRate\left (e_{i}\right)=\frac{top- bottom}{1+e^{- S\cdot \left (e_{i}- dock_{50}\right)}}+bottom$$

This function is defined by four parameters: (1) top: the maximum hit rate; (2) dock50: the docking score at which the hit rate is half of the top value (in kcal mol⁻¹); (3) S: the slope of the curve, calculated as S=slope × 4/top, where slope is the percentage change in hit rate at dock50 per kcal mol⁻¹; (4) bottom: the minimum hit rate, fixed at 0. To define prior distributions, we analyzed OR hit rates for *D. melanogaster* (Hallem and Carlson, 2006), *A. gambiae* (Carey et al., 2010), and *S. littorali* (de Fouchier et al., 2017) across eight docking energy intervals and used this empirical data to derive posterior distributions of the model parameters. We specified independent Bayesian priors for each parameter: P(top)~beta (*α*=20, *β*=80), P(dock50)~normal (μ = –10, σ=15), and P(S)~normal (μ=–0.6, σ=0.1; Figure 5—figure supplement 2B to D). Sampling from the prior distributions was performed using Hamiltonian Monte Carlo implemented in Stan, with parameters: ‘num_samples = 100000’, ‘num_chains = 4’, ‘num_warmup = 50000’, and ‘max_depth = 12’.

### Docking-profile similarity network construction and community labeling

We first calculated the Pearson correlation coefficients (PCCs) for docking energies between all OR pairs across the VOC library. ORs were then ranked by the number of VOCs meeting the docking-score threshold, and a greedy clustering algorithm was applied to pre-cluster ORs with high docking-profile similarity (PCCs ≥0.6), resulting in 6299 nodes. The edge weight between nodes was defined as the average PCC of all OR pairs. The docking-profile similarity network was then constructed using the ‘trunk–branch’ strategy. Due to the complexity of OR functional divergence, our strategy yielded a modularity value that deviated by 0.1 from the theoretical Louvain modularity. To ensure accurate community assignment, we adopted the Louvain algorithm for final community detection. A PCC threshold of 0.48 was selected as the cutoff for defining the final docking-profile similarity network and OR docking-profile communities. The network was visualized using the organic layout algorithm in Cytoscape (Shannon et al., 2003).

Then, we assigned a VOC docking breadth label to each docking-profile community. First, we counted the number of VOCs with each functional group that were with docking scores below −8 kcal/mol in each docking-profile community. These counts were then compared against the total number of ORs recognizing those VOCs across all ORs, using Bonferroni-corrected post hoc tests. If a community showed a significantly higher or lower recognition count, it was considered to have a higher or lower docking-derived VOC count for that functional group. The results were normalized by the average VOC count to derive a docking-profile breadth score for each dPC. *D. melanogaster* ORs were mapped to dPCs to evaluate the consistency between dPC labels and experimental response data. For each VOC functional group, ORs were assigned positive or negative labels according to their dPC labels, and these labels were compared with response counts from the *Drosophila* functional matrix to generate ROC curves and AUC values.

## Data Availability

The data supporting the findings of this study are available for download from the figshare repository at https://doi.org/10.6084/m9.figshare.30541190. All custom scripts and R code used for bioinformatics filtering and similarity network construction are available at GitHub (https://github.com/zhangtm0309/Code copy archived at Zhang, 2026) and archived on Zenodo (DOI: https://doi.org/10.5281/zenodo.18459276). The following datasets were generated: LuH 2025Class-wide sequence, structural, and docking-profile diversity of insect odorant receptors and structural specialization of Orcofigshare10.6084/m9.figshare.30541190PMC1373052442974913 LuH 2026code and datafigshare10.6084/m9.figshare.31230736 ZhangT 2026zhangtm0309/Code: code and Calcium imaging rawdataZenodo10.5281/zenodo.18459276 The following previously published datasets were used: The i5k Initiative 2018Genome assembly Mhra_1.0NCBI AssemblyGCA_003456935.1 BrandP RobertsonHM LinW PothulaR KlingemanWE Jurat-FuentesJL JohnsonBR 2019Data from: The origin of the odorant receptor gene family in insectsDryad Digital Repository10.5061/dryad.p2t8170PMC608094830063003 BCM-HGSC 2017Genome assembly Lful_2.0NCBI AssemblyGCA_000376725.2 Chinese Academy of Agricultural Sciences 2021Genome assembly CAAS_Pfla_1.0NCBI AssemblyGCA_020796165.1 WELLCOME SANGER INSTITUTE 2022Genome assembly ioPlaPenn1.1NCBI AssemblyGCA_933228895.1 The i5k Initiative 2017Genome assembly Edan_2.0NCBI AssemblyGCA_000507165.2 WELLCOME SANGER INSTITUTE 2021Genome assembly ipNemPict2.1NCBI AssemblyGCA_921293315.1 WELLCOME SANGER INSTITUTE 2022Genome assembly ipLeuNigr2.1NCBI AssemblyGCA_934045905.1 China Agricultural University 2022Genome assembly CAU_Lmig_1.0NCBI AssemblyGCA_026315105.1 Texas A&M University 2021Genome assembly TAMU-RSper-2021NCBI AssemblyGCA_021186555.1 WELLCOME SANGER INSTITUTE 2022Genome assembly ihAcaHaem1.1NCBI AssemblyGCA_930367205.1 University of Georgia 2022Genome assembly UGA_ACOO_1.1NCBI AssemblyGCA_023614345.1 The University of Texas at Austin 2019Genome assembly pea_aphid_22Mar2018_4r6ur_v2NCBI AssemblyGCF_005508785.2 China Agricultural University 2021Genome assembly ASM2088223v1NCBI AssemblyGCA_020882235.1 Key Laboratory of Forest Disaster Warning and Control of Yunnan Province 2021Genome assembly ASM2046615v1NCBI AssemblyGCA_020466155.1 Zhejiang University 2021Genome assembly Schi1.0NCBI AssemblyGCA_019022885.1 USDA-ARS 2022Genome assembly USDA_Piezodorus_guildinii_Assembly_v1.1NCBI AssemblyGCA_023052935.1 China Agricutural University 2022Genome assembly Lbru_1.0NCBI AssemblyGCA_023512825.1 WELLCOME SANGER INSTITUTE 2021Genome assembly iyAncNigr1.1NCBI AssemblyGCA_916049575.1 University of Copenhagen 2022Genome assembly ASM2471352v1NCBI AssemblyGCA_024713525.1 Zhejiang university 2021Genome assembly Gfla_zju_1.0NCBI AssemblyGCA_018340375.1 WELLCOME SANGER INSTITUTE 2022Genome assembly iyTipFemo1.1NCBI AssemblyGCA_944319695.1 WELLCOME SANGER INSTITUTE 2022Genome assembly iyRhoChlo1.1NCBI AssemblyGCA_944452935.1 Zhejiang University 2021Genome assembly ZJU_Trem_1.0NCBI AssemblyGCA_020615435.1 Nankai University 2021Genome assembly ASM1890698v1NCBI AssemblyGCA_018906985.1 WELLCOME SANGER INSTITUTE 2021Genome assembly iyAnoNige1.1NCBI AssemblyGCA_914767735.1 Sun Yat-sen University 2022Genome assembly ASM2257598v1NCBI AssemblyGCA_022575985.1 WELLCOME SANGER INSTITUTE 2022Genome assembly iyMacEuro1.2NCBI AssemblyGCA_916610135.2 Institute of Zoology, Chinese Academy of Sciences 2022Genome assembly ASM2240512v1NCBI AssemblyGCA_022405125.1 USDA-ARS 2021Genome assembly ASM1945775v1NCBI AssemblyGCA_019457755.1 WELLCOME SANGER INSTITUTE 2022Genome assembly iySelTumu1.3NCBI AssemblyGCA_913789895.3 University of Lausanne 2021Genome assembly UNIL_Sinv_3.0NCBI AssemblyGCA_016802725.1 Institute of Plant Physiology and Ecology SIBS CAS 2020Genome assembly ASM1547642v1NCBI AssemblyGCA_015476425.1 National Coffee Research Center - CENICAFE 2022Genome assembly Cenicafe_Pcof_1.1NCBI AssemblyGCA_024137745.1 United States Department of Agriculture 2021Genome assembly iyDipSimi1.1NCBI AssemblyGCA_021155765.1 WELLCOME SANGER INSTITUTE 2022Genome assembly iyPemLugu1.1NCBI AssemblyGCA_933228935.1 Institute of Zoology, Chinese Academy of Sciences 2020Genome assembly ASM1312311v1NCBI AssemblyGCA_013123115.1 IRBI-UMR 7261 CNRS - University of Tours 2021Genome assembly CoteBio_CtyphaeNCBI AssemblyGCA_013202065.2 National Coffee Research Center - CENICAFE 2022Genome assembly Cenicafe_Pnas_1.1NCBI AssemblyGCA_024137725.1 WELLCOME SANGER INSTITUTE 2021Genome assembly iyNomFabr1.1NCBI AssemblyGCA_907165295.1 WELLCOME SANGER INSTITUTE 2022Genome assembly iyAndDors1.1NCBI AssemblyGCA_929108735.1 Zhejiang University 2022Genome assembly NIG_curated_v1.5.1NCBI AssemblyGCA_024320075.1 WELLCOME SANGER INSTITUTE 2021Genome assembly inChrCarn1.1NCBI AssemblyGCA_905475395.1 Rudjer Boskovic Institute 2022Genome assembly Tfree1.0NCBI AssemblyGCA_939628115.1 USDA-ARS Ag100Pest 2023Genome assembly icTriCast1.1NCBI AssemblyGCA_031307605.1 WELLCOME SANGER INSTITUTE 2022Genome assembly icPhiCogn1.1NCBI AssemblyGCA_932526585.1 WELLCOME SANGER INSTITUTE 2022Genome assembly icSilAtra2.1NCBI AssemblyGCA_944588485.1 The University of Queensland 2021Genome assembly S4-7k-2v3NCBI AssemblyGCA_020654165.1 WELLCOME SANGER INSTITUTE 2022Genome assembly icPyrSerr1.2NCBI AssemblyGCA_905333025.2 ROTHAMSTED RESEARCH 2022Genome assembly PGI_MELIAE_v1NCBI AssemblyGCA_921294245.1 Northwestern Polytechnical University 2020Genome assembly ASM1336807v1NCBI AssemblyGCA_013368075.1 WELLCOME SANGER INSTITUTE 2022Genome assembly icPolCerv1.1NCBI AssemblyGCA_935413205.1 WELLCOME SANGER INSTITUTE 2021Genome assembly icHarAxyr1.1NCBI AssemblyGCA_914767665.1 ROTHAMSTED RESEARCH 2022Genome assembly PGI_DIABVI_V1NCBI AssemblyGCA_917563875.1 WELLCOME SANGER INSTITUTE 2022Genome assembly icLepMacu1.1NCBI AssemblyGCA_936432065.1 WELLCOME SANGER INSTITUTE 2022Genome assembly icPteMadi1.2NCBI AssemblyGCA_911728475.2 WELLCOME SANGER INSTITUTE 2021Genome assembly icRhaFulv1.1NCBI AssemblyGCA_905340355.1 WELLCOME SANGER INSTITUTE 2021Genome assembly icApoCory1.1NCBI AssemblyGCA_911728435.1 China Agricultural University 2019Genome assembly ASM897352v1NCBI AssemblyGCA_008973525.1 WELLCOME SANGER INSTITUTE 2022Genome assembly iiLimLuna2.2NCBI AssemblyGCA_917563855.2 Florida Museum of Natural History 2022Genome assembly ASM2284056v1NCBI AssemblyGCA_022840565.1 LOEWE Centre for Translational Biodiversity Genomics 2022Genome assembly ASM2265174v1NCBI AssemblyGCA_022651745.1 LOEWE Centre for Translational Biodiversity Genomics 2022Genome assembly ASM2260657v1NCBI AssemblyGCA_022606575.1 LOEWE Centre for Translational Biodiversity Genomics 2022Rhyacophila brunnea genome assembly ASM2249500v1NCBI AssemblyGCA_022495005.1 Florida Museum of Natural History 2022Genome assembly ASM2211383v1NCBI AssemblyGCA_022113835.1 WELLCOME SANGER INSTITUTE 2021Genome assembly ilZygFili1.2NCBI AssemblyGCA_907165275.2 WELLCOME SANGER INSTITUTE 2021Genome assembly ilYpsScab1.1NCBI AssemblyGCA_910592155.1 WELLCOME SANGER INSTITUTE 2022Genome assembly ilYpoSedl1.1NCBI AssemblyGCA_934045075.1 WELLCOME SANGER INSTITUTE 2021Genome assembly ilHedSali1.2NCBI AssemblyGCA_905404275.2 WELLCOME SANGER INSTITUTE 2021Genome assembly ilTinTrin1.1NCBI AssemblyGCA_905220615.1 WELLCOME SANGER INSTITUTE 2021Genome assembly ilMimTili1.1NCBI AssemblyGCA_905332985.1 WELLCOME SANGER INSTITUTE 2021Genome assembly ilSesApif2.1NCBI AssemblyGCA_914767545.1 Gakushuin University 2020Genome assembly Sr_UT_JL_v1.0NCBI AssemblyGCA_014132275.1 WELLCOME SANGER INSTITUTE 2022Genome assembly ilAcrSuav1.1NCBI AssemblyGCA_943193695.1 WELLCOME SANGER INSTITUTE 2021Genome assembly ilMarLuna1.1NCBI AssemblyGCA_923062675.1 University of Adelaide 2021Genome assembly PxLV.1NCBI AssemblyGCA_019096205.1 Wellcome Sanger Institute 2021Genome assembly ilPieBrab1.1NCBI AssemblyGCA_905147105.1 Stockholm University 2021Genome assembly Parnassius_apolloNCBI AssemblyGCA_907164705.1 WELLCOME SANGER INSTITUTE 2021Genome assembly ilFurFurc1.1NCBI AssemblyGCA_911728495.1 WELLCOME SANGER INSTITUTE 2022Genome assembly ilMegAlbu1.1NCBI AssemblyGCA_936450015.1 WELLCOME SANGER INSTITUTE 2022Genome assembly ilAteCent1.3NCBI AssemblyGCA_905333075.3 University of Florida 2021Genome assembly ASM2038319v1NCBI AssemblyGCA_020383195.1 Wellcome Sanger Institute 2021Genome assembly ilAriAges1.1NCBI AssemblyGCA_905147365.1 Southwest Forestry University 2021Genome assembly ASM1992509v1NCBI AssemblyGCA_019925095.1 WELLCOME SANGER INSTITUTE 2022Genome assembly ilPyrMalv3.2 alternate haplotypeNCBI AssemblyGCA_911387725.2 WELLCOME SANGER INSTITUTE 2021Genome assembly ilCroElin1.1NCBI AssemblyGCA_907269065.1 Wellcome Sanger Institute 2022Genome assembly ilLymMona1.2NCBI AssemblyGCA_905163515.2 WELLCOME SANGER INSTITUTE 2021Genome assembly ilHabPyri1.1NCBI AssemblyGCA_907165245.1 WELLCOME SANGER INSTITUTE 2022Genome assembly ilCarQuer1.2NCBI AssemblyGCA_910589575.2 WELLCOME SANGER INSTITUTE 2022Genome assembly ilCalPalu1.1NCBI AssemblyGCA_927399485.1 WELLCOME SANGER INSTITUTE 2021Genome assembly ilZeuPyri1.1NCBI AssemblyGCA_907165235.1 Beijing Academy of Agriculture and Forestry Sciences 2020Genome assembly ASM1460749v2NCBI AssemblyGCA_014607495.2 Graduate School of Agricultural and Life Sciences, The University of Tokyo 2020Genome assembly Bmori_2016v1.0NCBI AssemblyGCA_014905235.2 WELLCOME SANGER INSTITUTE 2021Genome assembly ilBlaAdus2.1NCBI AssemblyGCA_907269095.1 WELLCOME SANGER INSTITUTE 2021Genome assembly ilEupTran1.1NCBI AssemblyGCA_914767815.1 Chinese Academy of Inspection and Quarantine 2021Genome assembly ASM2028386v1NCBI AssemblyGCA_020283865.1 WELLCOME SANGER INSTITUTE 2022Genome assembly idNepFlae1.1NCBI AssemblyGCA_932526605.1 WELLCOME SANGER INSTITUTE 2021Genome assembly idCheVulp2.1NCBI AssemblyGCA_916610125.1 CAM 2020Genome assembly iHerIll2.2.curated.20191125NCBI AssemblyGCA_905115235.1 WELLCOME SANGER INSTITUTE 2022Genome assembly idPheCory1.1NCBI AssemblyGCA_943735915.1 Brown University 2020Genome assembly BU_Bcop_v1NCBI AssemblyGCA_014529535.1 WELLCOME SANGER INSTITUTE 2022Genome assembly idSarRose1.1NCBI AssemblyGCA_930367235.1 WELLCOME SANGER INSTITUTE 2022Genome assembly idStoLuna1.1NCBI AssemblyGCA_933228675.1 WELLCOME SANGER INSTITUTE 2022Genome assembly idPolAngu1.1NCBI AssemblyGCA_930367215.1 Institute of Zoology 2022Genome assembly ASM2308958v1NCBI AssemblyGCA_023089585.1 Stanford University 2021Genome assembly ASM1890436v1NCBI AssemblyGCA_018904365.1 WELLCOME SANGER INSTITUTE 2022Genome assembly idAnoGambNW_F1_1NCBI AssemblyGCA_943734735.1 WELLCOME SANGER INSTITUTE 2022Genome assembly idMyoTess1.1NCBI AssemblyGCA_943737955.1 WELLCOME SANGER INSTITUTE 2021Genome assembly idCluTigr1.1NCBI AssemblyGCA_920105625.1 Hunan Agricultural University 2021Genome assembly Cory_1.0NCBI AssemblyGCA_020466095.1 Tianjin Normal University 2021Genome assembly ASM1839793v1NCBI AssemblyGCA_018397935.1 WELLCOME SANGER INSTITUTE 2022Genome assembly idBelPand1.2NCBI AssemblyGCA_916048285.2 WELLCOME SANGER INSTITUTE 2022Genome assembly idBomDisc1.1NCBI AssemblyGCA_939192795.1 WELLCOME SANGER INSTITUTE 2021Genome assembly idBibMarc1.1NCBI AssemblyGCA_910594885.1 WELLCOME SANGER INSTITUTE 2022Genome assembly idMacAtri3.1NCBI AssemblyGCA_933228815.1

## Additional files

Supplementary file 1. Benchmarking of insect odorant receptor annotation. Tables A-C assess the completeness and reliability of the odorant receptor annotation and structural-filtering workflow. Table A compares receptor counts with published repertoires. Table B summarizes correspondence between predicted receptors and curated reference annotations. Table C examines the genomic locations of selected *Bombyx mori* candidates to assess potential exon-merging artifacts in tandem receptor regions.

MDAR checklist

## Appendix 1

### Benchmarking of OR annotation completeness and structural filtering

To evaluate the completeness and reliability of the OR annotation workflow, we benchmarked the automated annotation and structural-filtering pipeline against curated OR repertoires from three well-studied insect species: *Drosophila melanogaster*, *Anopheles gambiae*, and *Bombyx mori*. These species were selected because their OR repertoires have been extensively annotated and are among the best curated datasets available for comparison. The benchmark was designed to assess three aspects of the workflow: (i) recovery of previously reported ORs by the automated annotation pipeline, (ii) the effect of structural filtering on OR model quality, and (iii) whether candidate ORs in tandem-rich regions could reflect exon-merging artifacts.

For each benchmark species, we recorded the number of ORs recovered before structural filtering, the number retained after structural filtering, and the correspondence between retained candidates and literature-annotated ORs. Candidate ORs were matched to published ORs based on sequence similarity (Supplementary file 1B). When discrepancies occurred, the corresponding sequences and predicted structures were manually inspected. For *Bombyx mori* candidates that did not correspond one-to-one to literature-annotated ORs, we further examined genomic locations to evaluate whether they may have resulted from exon merging among adjacent tandem ORs (Supplementary file 1C). Candidates located on different chromosomes or outside the same tandem region as their closest literature ORs were not considered likely exon-merging artifacts.

For *D. melanogaster*, the accepted repertoire contains 60 OR genes and 65 protein products, including splice variants. At the gene level, the automated workflow recovered most benchmark ORs before structural filtering, but Or47b and Or98b were not annotated. After structural filtering, fragmented or structurally incomplete ORs were removed, leaving 56 ORs that otherwise correspond one-to-one with the benchmark ORs. Two ORs that had been annotated before filtering, DmOr59a and DmOr85e, were removed by the structural filter because DmOr59a was fragmented and DmOr85e lacked TM7 in the predicted structural model.

The published *A. gambiae* OR repertoire contains 79 OR genes. The automated annotation workflow initially recovered 77 ORs, including 72 previously reported ORs. After removing fragmented ORs and five literature-annotated full-length ORs, 67 structurally intact ORs were retained. The retained ORs otherwise corresponded to literature annotations. Because five published full-length ORs were excluded by the structural filter, we further examined their predicted structures. AgOr52, AgOr47, and AgOr64 lacked one complete transmembrane region. AgOr58 and AgOr6 contained all expected transmembrane regions, but showed local deletions in part of the transmembrane regions. These results indicate that structural filtering can remove clearly incomplete ORs, but may also exclude a small number of genuine ORs with atypical or locally incomplete transmembrane-region features.

The published *B. mori* OR repertoire contains 66 OR genes, including two pseudogenes. The automated workflow initially annotated 103 OR candidates, including 60 literature-annotated ORs. After structural filtering, fragmented ORs and seven literature-annotated ORs were removed, and 64 structurally intact ORs were retained. Among these, 53 corresponded to literature-annotated ORs. Some retained candidates showed high sequence similarity to literature ORs, whereas others showed lower similarity and did not clearly correspond to previously reported ORs. To assess whether such candidates could result from exon-merging errors in tandem OR regions, we examined their genomic positions. Most candidates did not occur on the same chromosome or in the same tandem region as their closest literature-annotated ORs. Therefore, these candidates are unlikely to be simple exon-merging artifacts involving adjacent OR genes, and may represent previously unannotated OR candidates. Nevertheless, transcriptomic evidence or manual gene-model curation will be needed to confirm these candidates.

Because previously known OR sequences from some benchmark species were included in the query database, we next tested whether the presence of conspecific queries systematically influenced the final inferred OR repertoire size. We divided the benchmark species into two groups: those with conspecific OR sequences represented in the query database and those without conspecific queries. We detected no significant difference in OR annotation performance between the two groups (p=0.8, Figure 1—figure supplement 1B). Because *Anagrus nilaparvatae* showed an unusually large difference in OR number between the two studies, we further performed a sensitivity analysis excluding this species, and the conclusion remained unchanged (p=0.4, Figure 1—figure supplement 1C). Therefore, among species for which comparable genome-based reference repertoires are currently available, we found no evidence that the presence or absence of conspecific ORs in the query database systematically affects the final inferred OR repertoire size.

To extend the benchmarking beyond these three detailed case studies, we compared the final numbers of structurally intact ORs with published repertoire counts from additional insect species (Supplementary file 1A). For most species with comparable genome-based reference repertoires, the number of ORs retained in this study exceeded 70% of the published count and showed generally good agreement in repertoire size. *Diabrotica virgifera virgifera* was an exception: a previous manual annotation reported 124 complete ORs, whereas our pipeline retained 78 structurally intact ORs, approximately 63% of the published count. This discrepancy likely reflects the challenges posed by the large, repeat-rich genome of *D. virgifera virgifera*, in which repetitive sequences and complex OR loci can complicate automated gene prediction and locus reconstruction. The previous species-specific annotation incorporated transcriptomic evidence and locus-by-locus manual curation, which can help resolve gene models in complex genomic regions. Therefore, transcriptome-assisted manual annotation may be required to obtain more comprehensive OR repertoires for species with large, repeat-rich, or otherwise complex genomes.

Overall, the benchmarking analysis indicates that the automated annotation workflow recovers most known OR repertoires in well-annotated species and that structural filtering improves dataset reliability for downstream structural comparisons and docking-profile analyses. The final dataset was deliberately designed as a conservative, high-confidence set of structurally intact ORs rather than the broadest possible species-level gene catalog. Accordingly, lower counts relative to some published repertoires are an expected consequence of uniformly excluding fragments, pseudogenes, and candidates that do not meet the structural inclusion criteria. To evaluate whether this conservative filtering influenced the ecological analyses, we repeated the phylogenetic comparative analyses using the larger of our structurally intact OR count and the corresponding published count. The main associations between OR repertoire size and ecological traits remained unchanged (Figure 1—figure supplement 1D–G), indicating that the filtering strategy did not affect our conclusions.

## Appendix 2

### Hit-rate benchmark for docking-score threshold selection

To evaluate whether the docking-score threshold was driven by any single functional dataset, we performed hit-rate analyses using experimentally characterized OR–VOC pairs from multiple insect groups.

When *D. melanogaster*, *Anopheles gambiae*, and Lepidoptera datasets were analyzed separately, they showed similar docking score-hit rate relationships. In all three datasets, experimentally responsive OR–VOC pairs were enriched as docking scores became more negative. The hit-rate plateau for these datasets was approximately 23%, and the corresponding docking-score threshold was around –8 kcal/mol. This result is consistent with the threshold used in the main analysis and indicates that the –8 kcal/mol cutoff was not driven by a single species or dataset.

We also included *Locusta migratoria* OR functional data as an independent sensitivity analysis. The locust dataset differed from the other datasets in two important ways. First, the experimental positive rate was low, approximately 5.2%. Second, previously characterized locust ORs are mostly narrowly tuned receptors.

Consistent with these properties, the locust dataset showed lower overall hit rates than the *Drosophila*, *Anopheles*, and Lepidoptera datasets. In most docking-score intervals, the locust hit rate remained below 10% (Figure 5—figure supplement 1G). When the locust dataset was combined with the other three datasets, the overall hit-rate plateau decreased to approximately 18%, and the corresponding docking-score threshold shifted to approximately –11 kcal/mol (Figure 5—figure supplement 1H).

The locust result indicates that OR tuning breadth and the experimental positive rate of a dataset can influence the relationship between docking score and hit rate. For datasets dominated by narrowly tuned ORs and low experimental positive rates, a stricter docking-score threshold may be required to achieve stronger enrichment of experimentally responsive OR–VOC pairs.

However, the goal of the present study was to establish a unified empirical threshold for large-scale comparison across thousands of ORs and a broad VOC library, rather than to optimize separate thresholds for individual lineages. Therefore, the –8 kcal/mol threshold derived from the *Drosophila*, *Anopheles*, and Lepidoptera datasets was retained for the main analysis, whereas the locust result was treated as a sensitivity analysis and used to define the limitation of applying a single cutoff across all insect groups.

The hit-rate benchmark supports the use of docking scores for repertoire-level enrichment and comparative analysis. Nevertheless, the plateau hit rate remains limited and is not sufficient to assign definitive ligands or experimentally validated response spectra to individual ORs.

Therefore, docking results in this study should be interpreted as docking-derived binding potential. They provide a high-throughput, internally comparable framework for identifying repertoire-level trends and candidate OR–VOC relationships for future testing, but specific receptor-ligand pairs require validation by heterologous expression, electrophysiology, calcium imaging, genetic perturbation, or behavioral assays.
