# Supplementary material for: Class-wide sequence, structural, and docking-profile diversity of insect odorant receptors and structural specialization of Orco
Source: eLife. 2026 Nov 3;15:RP110877. doi: 10.7554/eLife.110877 (PMC13730524; doi:10.7554/eLife.110877)
Supplement: Peer review history

# Editor report: eLife Assessment

**DOI:** 10.7554/eLife.110877.4.sa0

**Authors:** Virginie Courtier-Orgogozo

**Affiliations:** CNRS - Universite Paris Cite France

**Keywords:** Important, Solid

This **important** large-scale comparative study of odorant receptor (OR) genes across more than 100 insect species, combining sequence- and structure-based approaches, represents a significant contribution in the exploration of the evolution of this large gene family involved in the detection of odorant signals by olfactory neurons. Most notably, the authors uncover a structural feature unique to the odorant receptor co-receptor Orco that reduces ligand binding affinity. From this **solid** data emerges a general integrative framework to apprehend the diversity and evolution of odorant receptor genes in insects.

# Referee report: Reviewer #1 (Public review)

**DOI:** 10.7554/eLife.110877.4.sa1

[Editors' note: this version has been assessed by the Reviewing Editor without further input from the original reviewers. All the reviewers' comments have been adequately addressed and taken into account.]

Objectives of the study and impact of the work

The authors of this article primarily aim to reconstruct the evolutionary history of the insect odorant receptor (OR) family, which is responsible for the detection of odorant signals by olfactory neurons. Due to the lack of phylogenetic signal present in the sequences of this multigene family, which evolves very rapidly, phylogenetic analyses have so far never made it possible to precisely retrace how ORs diversified prior to the appearance of present-day insect orders, and what the drivers of this diversification were. For example, one may suspect that the adaptation of ORs to odors emitted by plants constituted a critical step in insect evolution during the "angiosperm terrestrial revolution," which occurred at the end of the Cretaceous, but nothing currently allows this to be asserted.

There are very nice examples, notably in drosophilids, derived from comparisons between closely related species and documenting mechanisms of OR adaptation to certain signals. However, what the authors attempt to do in this work is to produce a macroevolutionary analysis at the scale of insects as a whole, based almost exclusively on bioinformatic analyses. To do this, they annotated OR genes in about one hundred insect species and developed pipelines for analyzing sequence similarity, structural similarity, and functional similarity, the latter being estimated through a molecular docking approach. An important element in the evolution of insect ORs is the appearance of a unique co-receptor, called Orco, which appears to be an OR that has lost the ability to bind odorants. In addition to the large-scale bioinformatic analysis, the authors also aim to explore more specifically the factors that favored the emergence of Orco and the selective advantage conferred by the existence of OR-Orco complexes.

Given the importance of odorant receptors in insect biology and in their adaptation to different environments and lifestyles, retracing their evolutionary history is indeed a major question in evolutionary biology. In principle, this type of work therefore has the potential to become a reference in the field and to provide a basis for significant scientific advances.

Major strengths and weaknesses

The sampling chosen for collecting OR sequences is very impressive, with more than 100 insect families represented, covering most of the major orders. This sampling appears appropriate for the question being addressed. The analysis pipeline used to collect the sequences makes sense, relying on homology-based annotation tools coupled with a structure-based filter. Nevertheless, one can note aberrant numbers of ORs for certain species (much lower than reality). A lower number of OR genes is somewhat expected, as the authors chose to apply a fairly stringent filter on sequence quality (based on predicted 3D structure), which reduces the number from 14,000 to 9,000. This choice seems logical given the subsequent use of these data, but it inevitably leads to data loss. However, the low number of genes also results from the fact that the pipeline did not function correctly for all genomes.

In the revised version of the manuscript, the authors included a benchmarking step, which is a good point. They compared their OR gene annotations with previous reports in the same species. Unfortunately, this comparison is irrelevant because the chosen reference OR repertoires are actually a mix of transcriptome and genome annotations. Furthermore, comparisons with entire OR gene repertoires essentially demonstrate that their annotation was of good quality for species in which OR sequences were already present in the query OR database, but poor for species in which they were not. Therefore, these supplementary analyses made by the authors are not particularly in favor of an overall good quality of OR gene repertoires in the >110 species studied. The fact that some OR genes may be missing and that the total number may not be exact for each species is not prohibitive for studying the evolution of the family on a broad scale. However, it does call into question the correlation between the number of ORs and lifestyles and diets.

From the dataset collected, the authors attempted to categorize ORs in several ways, starting with the reconstruction of sequence similarity networks. The approach is interesting, but fails to reveal homology relationships between ORs from species belonging to different insect orders. So, it is unclear what the advantage of this approach is compared with the "classical" phylogenetic approach.

The clustering based on structure also leads to the identification of a majority of "order-specific" clusters, which does not provide major insights into the evolution of ORs. However, the authors highlight a group of ORs in flies that appear to possess an unusual intracellular region, as well as a cluster of OR shared across many insect orders that exhibit a larger binding cavity. This is really interesting, although more relevant to OR structure than to their evolution.

The analysis of structural diversity then leads the authors to focus on the Orco co-receptors, which are characterized by modifications of the binding pocket and the appearance of an extracellular loop that could explain the loss of the ability to bind odorant molecules. This part, which relies on in vitro experiments, is interesting and constitutes the most striking result of this study, which could in itself have been the subject of a separate manuscript.

The rest of the manuscript is based on the prediction of OR response spectra using molecular docking. The work that has been carried out is extremely substantial, and the objective of linking clusters based on sequence similarity or 3D structural similarity with functional categories is entirely relevant. The docking score threshold used was chosen thoughtfully, which is very good, and according to the calculation performed should ensure a true positive rate of more than 20%, which is excellent in such a docking analysis. But in the absence of functional validation, this 20% true positive rate is not sufficient to extrapolate OR function, and docking-derived binding breadth measures used in the remaining of the manuscript have to be taken with caution, as acknowledged by the authors themselves. Consequently, the chance that results of this part of the work will enlighten the evolution of OR on a broad scale is low. For example, the fact that insect lineages that emerged after the Permian-Triassic extinction have more broadly-tuned OR is an interesting observation, yet remains highly speculative.

In summary, despite the large number of analyses performed, the authors do not really succeed in achieving the stated objective of reconstructing the evolutionary history of insect ORs, and the results obtained do not strongly support all the conclusions regarding the links between OR repertoires and environment or lifestyle.

# Referee report: Reviewer #2 (Public review)

**DOI:** 10.7554/eLife.110877.4.sa2

The remarkable evolvability of the olfactory system enables animals to rapidly adapt to dynamic and chemically complex environments. Over the past two decades, substantial effort has been devoted to uncovering the evolutionary principles that drive the diversification of odorant receptors (ORs), yielding key insights into the forces shaping their striking variability in both vertebrates and insects. In this manuscript, Zhang and colleagues analyze the OR repertoires of over 100 insect species, leveraging sequence and structural similarity to infer patterns of gene family evolution within this diverse and ecologically important clade. By integrating sequence-based and structure-based comparisons, their study builds on a compelling and recently emerging line of research made possible by the advent of AlphaFold, which has previously clarified the phylogenetic relationship between insect Ors and the gustatory receptor gene family and revealed the unexpectedly deep evolutionary origins of this ancient structural fold.

Applying this approach to a large set of ORs derived from species throughout the insect phylogeny, the authors confirm many previously reported patterns of OR evolution. More importantly, with their large-scale approach, the authors found new structural features of different Or clades, resulting in intriguing testable hypotheses about Or evolution that have the potential to support future directions in studying this important gene family. For example, the authors identify a structural feature mostly unique to the OR co-receptor ORco, a beta-sheet in EL2, which they functionally show reduces odorant binding affinity - a key aspect of ORco, which does not bind ligands in the ancestral ligand-biding site. This is a particularly strong part of the manuscript, since the authors support their in silico-derived hypothesis with functional data.

In an attempt to assess the relationship between sequence identity and structure on one hand and function on the other, the authors perform an in-silico structure prediction and chemical docking analysis. While not suitable to assess the actual function of individual receptors without functional testing, this strategy revealed potential receptor clades with differences in the number and type of docked chemicals. As a response to the previous reviews, the authors now changed their writing to refer to these results as 'docking-derived function' instead of just 'function'. While I appreciate that the authors changed their wording, this semantic change still can be read as referring to docking-derived predictions as conclusions of actual biological function. However, this is not the case. Any functional claims based solely on the docking approach need to be interpreted with caution, as in silico-only approaches are currently not well enough predictors for actual receptor function. Accordingly, I strongly recommend to remove the parts that connect receptor function to structure or ecology.

Assessing their docking analyses with respect to species ecology, the authors identify correlations suggesting that Or repertoires might follow predictable evolutionary trajectories broadly aligned with species ecology. These are interesting hypotheses. However, due to the inability to predict actual receptor function the author's claims about ecology-function relationships are not well supported. Accordingly, the authors should change the respective parts of the manuscript in way that it is clear these are currently untested hypotheses. This includes adjusting the title, since the work does not find any experimental support for ecological tuning.

I believe that future studies combining functional testing with an explicit evo-eco framework could benefit from this work and the explicit hypotheses it generates. But the authors should be careful to not overstate their in-silico derived results.

# Author comment: Author response

**DOI:** 10.7554/eLife.110877.4.sa3

**Authors:** Tianmin Zhang, Xuanxiao Yang, Yufang Fu, Wei Xue, Yifeng Zhang, Suyang Duan, Yangming Yin, Yi Guo, Chenxi Gao, Yang Liu, Gang Li, Chang Xu, Huimeng Lu

**Affiliations:** Northwestern Polytechnical University xian China; Northwestern Polytechnical University xian China; Shaanxi Normal University Xi'an China; Northwestern Polytechnical University Xi'an China; Northwestern Polytechnical University Xi'an China; Northwestern Polytechnical University Xi'an China; Northwestern Polytechnical University Xi'an China; Northwestern Polytechnical University Xi'an China; Northwestern Polytechnical University Xi'an China; Shaanxi Normal University Xi'an China; Shaanxi Normal University Xi'an China; Shaanxi Normal University Xi'an China; Northwestern Polytechnical University Xi'an China

The following is the authors’ response to the previous reviews.

**Public Reviews:**

**Reviewer #1 (Public review):**

Objectives of the study and impact of the work

The authors of this article primarily aim to reconstruct the evolutionary history of the insect odorant receptor (OR) family, which is responsible for the detection of odorant signals by olfactory neurons. Due to the lack of phylogenetic signal present in the sequences of this multigene family, which evolves very rapidly, phylogenetic analyses have so far never made it possible to precisely retrace how ORs diversified prior to the appearance of present-day insect orders, and what the drivers of this diversification were. For example, one may suspect that the adaptation of ORs to odors emitted by plants constituted a critical step in insect evolution during the "angiosperm terrestrial revolution," which occurred at the end of the Cretaceous, but nothing currently allows this to be asserted.

There are very nice examples, notably in drosophilids, derived from comparisons between closely related species and documenting mechanisms of OR adaptation to certain signals. However, what the authors attempt to do in this work is to produce a macroevolutionary analysis at the scale of insects as a whole, based almost exclusively on bioinformatic analyses. To do this, they annotated OR genes in about one hundred insect species and developed pipelines for analyzing sequence similarity, structural similarity and functional similarity, the latter being estimated through a molecular docking approach. An important element in the evolution of insect ORs is the appearance of a unique co-receptor, called Orco, which appears to be an OR that has lost the ability to bind odorants. In addition to the large-scale bioinformatic analysis, the authors also aim to explore more specifically the factors that favored the emergence of Orco and the selective advantage conferred by the existence of OR-Orco complexes.

Given the importance of odorant receptors in insect biology and in their adaptation to different environments and lifestyles, retracing their evolutionary history is indeed a major question in evolutionary biology. In principle, this type of work therefore has the potential to become a reference in the field and to provide a basis for significant scientific advances.

Major strengths and weaknesses

The sampling chosen for collecting OR sequences is very impressive, with more than 100 insect families represented, covering most of the major orders. This sampling appears appropriate for the question being addressed. The analysis pipeline used to collect the sequences makes sense, relying on homology-based annotation tools coupled with a structure-based filter. Nevertheless, one can note aberrant numbers of ORs for certain species (much lower than reality). A lower number of OR genes is somewhat expected, as the authors chose to apply a fairly stringent filter on sequence quality (based on predicted 3D structure), which reduces the number from 14,000 to 9,000. This choice seems logical given the subsequent use of these data, but it inevitably leads to data loss. However, the low number of genes also results from the fact that the pipeline did not function correctly for all genomes.

In the revised version of the manuscript, the authors included a benchmarking step, which is a good point. They compared their OR gene annotations with previous reports in the same species. Unfortunately, this comparison is irrelevant because the chosen reference OR repertoires are actually a mix of transcriptome and genome annotations. Furthermore, comparisons with entire OR gene repertoires essentially demonstrate that their annotation was of good quality for species in which OR sequences were already present in the query OR database, but poor for species in which they were not. Therefore, these supplementary analyses made by the authors are not particularly in favor of an overall good quality of OR gene repertoires in the >110 species studied. The fact that some OR genes may be missing and that the total number may not be exact for each species is not prohibitive for studying the evolution of the family on a broad scale. However, it does call into question the correlation between the number of ORs and lifestyles and diets.

Thank you for this important comment. We agree that it is inappropriate to directly compare genome-based OR annotations with OR repertoires derived solely from transcriptomes, because transcriptome-only datasets generally do not capture the complete OR gene complement of a species. We therefore repeated the benchmarking analysis and collected, wherever available for the species included in this study, published OR repertoires based on genome annotation or genome annotation supported by transcriptomic evidence. Transcriptome-only OR datasets are no longer included in this comparison.

The updated benchmarking analysis shows that, for most species with comparable genome-based reference repertoires, the number of ORs identified in this study exceeds 70% of the previously reported number, with good agreement in OR repertoire size for most species (Table S1). *Diabrotica virgifera* is an exception: a previous manual annotation reported 124 complete ORs, whereas we identified 78, approximately two-thirds of the previously reported number. This result indicates that our automated annotation and stringent structural filtering pipeline may indeed underestimate OR repertoire size in some species. The large, repeat-rich genome of this species may further complicate gene prediction and the reconstruction of OR loci. We have therefore explicitly stated in the revised manuscript that incorporating transcriptomic evidence and manual curation may help recover more complete OR repertoires from such complex genomes.

Another notable outlier is *Anagrus nilaparvatae*. A previous study reported 88 ORs, whereas we identified 330. Examination of the methods used in that study revealed that its OR search relied primarily on homology searches using known OR sequences from two other hymenopteran species. Because insect ORs constitute a rapidly evolving gene family with pronounced lineage-specific expansions, a limited query set may fail to detect highly divergent or species-specific ORs. In comparison, our study used OR queries from a broader range of insects and a genome-level annotation pipeline specifically designed for insect ORs, which may have enabled the detection of more highly divergent candidate ORs. Because the protein sequences of the 88 ORs were not made available in the original study, we are currently unable to perform a one-to-one comparison to verify whether all of these ORs are included in our annotations.

You also noted that our better performance in species such as *Drosophila melanogaster*, *Anopheles gambiae*, and *Bombyx mori* might partly reflect the inclusion of known OR sequences from these species in the query database. We consider this an important and directly testable question. We therefore divided the benchmark species into two groups according to whether conspecific OR sequences were represented in the query database, and used ln(OR count_this study / OR count_previous) to quantify differences in OR counts relative to previous genome-based annotations. No significant difference was detected between the two groups (P = 0.841, Fig. S1 B). Given the unusually large difference in OR counts between the two studies for *A. nilaparvatae*, we also performed a sensitivity analysis excluding this species, which yielded the same conclusion (P = 0.413, Fig.S1 C). Thus, among the species for which comparable genome-based reference repertoires are currently available, we found no evidence that the inclusion of conspecific ORs in the query database systematically affected the final OR repertoire size. We have added this analysis to the supplementary material of the revised manuscript.

Finally, we agree that annotation biases in OR repertoire size may affect analyses of its correlations with ecological traits. We therefore repeated all relevant ecological sensitivity analyses using an updated OR count dataset, taking the larger of the OR counts from our study and the previous annotation for each species (Fig. S1 D- G). The main statistical results remained consistent with the original analyses: OR counts were still significantly associated with adult diet, larval diet, and habitat, whereas no significant association was detected with circadian rhythm. We therefore consider the statistical associations between OR counts and the main ecological variables to be reasonably robust within the range of annotation discrepancies detectable using the currently available genome-based benchmarks, although automated annotation and stringent structural filtering may underestimate OR counts in some species. The updated benchmarking analyses are described in Supplementary Text S1 and summarized in Table S1.

The following revision has been added (lines 103-107): “Benchmarking against curated OR repertoires showed that the automated pipeline recovered most previously reported ORs and that stringent structural filtering produced a high-confidence set of structurally intact receptors (Supplementary Text S1, Tables S1–S3, and fig. S1B–C). For most species with comparable genome-based references, retained OR counts exceeded 70% of published values.”

From the dataset collected, the authors attempted to categorize ORs in several ways, starting with the reconstruction of sequence similarity networks. The approach is interesting, but fails to reveal homology relationships between ORs from species belonging to different insect orders. So it is unclear what the advantage of this approach is compared with the "classical" phylogenetic approach.

Thank you for raising this important question. Our intention was not to replace classical phylogenetic analysis with sequence similarity networks. Rather, we used network-based approaches as a class-wide framework to classify ORs independently according to sequence similarity, structural similarity, and docking profiles, and then compared how these different classifications correspond across insect taxa and major macroevolutionary transitions.

For smaller gene families with limited sequence divergence, defining subfamilies based on gene trees is a well-established and highly effective approach, because phylogenetic trees can both identify subfamilies and reveal their evolutionary relationships. However, the insect OR superfamily exhibits pronounced birth-and-death evolution, with frequent gene duplications and losses across lineages, and comprises a vast number of highly divergent sequences. Consequently, when a single phylogenetic tree is constructed for the entire OR superfamily, deep nodes often lack sufficient support, and relationships among subfamilies may be difficult to resolve consistently. It is therefore challenging to obtain a stable and consistent classification of thousands of ORs into subfamilies across insects using a single gene tree alone. We therefore used sequence similarity networks as a complementary classification approach. Their main advantage is that they allow large datasets to be grouped according to sequence similarity among all ORs without requiring the resolution of every deep phylogenetic relationship. More importantly, similar network frameworks can be used to construct sequence similarity, structural similarity, and docking-profile similarity networks, allowing SeqC, StrC, and dPC to be compared directly within a common analytical framework. This is difficult to achieve using conventional gene trees alone and is the main reason for our choice of a network-based approach.

Sequence similarity networks alone cannot directly resolve homology relationships among ORs from different insect orders. During the previous revision, we therefore sought to link sequence communities to phylogenetic relationships. We conducted additional phylogenetic analyses of shared sequence communities containing multiple insect orders to search for potentially conserved OR lineages spanning different orders. However, among these shared communities, we identified only one conserved OR lineage spanning four insect orders with relatively clear phylogenetic support. We have added this result to the Supplementary Data S6. This finding further indicates that clearly identifiable orthology relationships spanning multiple orders are very limited among extant insect ORs.

The following revision has also been added (lines 150-154): “The SSN is not intended to replace phylogenetic reconstruction, but rather to provide a complementary framework for summarizing sequence relationships among thousands of highly divergent ORs at a class-wide scale. It allows major patterns of OR sequence similarity and diversification to be visualized more clearly across the entire dataset. Phylogenetic analyses can then be used to further resolve evolutionary relationships within specific sequence communities.”

The clustering based on structure also leads to the identification of a majority of "orderspecific" clusters, which does not provide major insights into the evolution of ORs. However, the authors highlight a group of ORs in flies that appear to possess an unusual intracellular region, as well as a cluster of OR shared across many insect orders that exhibit a larger binding cavity. This is really interesting, although more relevant to OR structure than to their evolution.

Thank you for this important comment. We agree that the previous version of the manuscript focused primarily on structural differences among structural communities and did not adequately explore their potential evolutionary implications. StrC23 is the most widely distributed structural community, spanning 12 insect orders and containing 46.3% of the ORs. We further found that its putative binding pockets were significantly larger than those of other ORs, which we previously interpreted as a potentially conserved structural pattern across taxa. Following your suggestion, we conducted additional analyses of the phylogenetic and orthology relationships within StrC23. The results showed that StrC23 does not correspond to a single, broadly conserved orthogroup spanning insect orders, but instead contains 156 distinct orthologous groups. Of these, 24 encompass more than 10 species. Only a few of these larger groups span multiple insect orders: one contains ORs from Hymenoptera, Diptera, and Trichoptera, and two contain ORs from both Lepidoptera and Trichoptera. The remaining groups show pronounced order specificity, including nine Hymenoptera-specific groups, eight Lepidoptera-specific groups, and four Diptera-specific groups.

These results indicate that the large putative binding pocket shared by StrC23 members cannot simply be attributed to a single conserved orthologous lineage spanning multiple orders, but is distributed across several phylogenetically independent OR lineages. This pattern may reflect convergent evolution toward similar structural features in different OR lineages, or the retention of an older structural feature in multiple OR lineages that subsequently underwent substantial sequence divergence. The current data do not allow us to distinguish conclusively between these two possibilities. We have added the phylogenetic and orthologous-group analyses of StrC23 to the revised manuscript and supplementary material to further clarify the evolutionary implications of this structural community shared across insect orders.

The following revision has been added (lines 208-218): “To further investigate the evolutionary basis of this broadly shared structural feature, we examined the phylogenetic and orthology relationships among StrC23 receptors. StrC23 was divided into 156 orthologous groups, 24 of which contained ORs from more than 10 species. However, only a few of these larger groups spanned multiple insect orders: one included ORs from Hymenoptera, Diptera, and Trichoptera, while two included ORs from both Lepidoptera and Trichoptera (Supplementary Data S6). Thus, StrC23 does not represent a single conserved cross-order orthologous lineage, but instead comprises multiple phylogenetically distinct OR lineages. Its shared large-pocket architecture therefore cannot be explained solely by conservation of a single orthologous lineage and may reflect either structural convergence or retention of an ancient structural feature followed by substantial sequence divergence.”

The analysis of structural diversity then leads the authors to focus on the Orco co-receptors, which are characterized by modifications of the binding pocket and the appearance of an extracellular loop that could explain the loss of the ability to bind odorant molecules. This part, which relies on in vitro experiments, is interesting and constitutes the most striking result of this study, which could in itself have been the subject of a separate manuscript.

We thank the reviewer for the positive assessment of this part of our work.

The rest of the manuscript is based on the prediction of OR response spectra using molecular docking. The work that has been carried out is extremely substantial, and the objective of linking clusters based on sequence similarity or 3D structural similarity with functional categories is entirely relevant. The docking score threshold used was chosen thoughtfully, which is very good, and according to the calculation performed should ensure a true positive rate of more than 20%, which is excellent in such a docking analysis. But in the absence of functional validation, this 20% true positive rate is not sufficient to extrapolate OR function, and docking-derived binding breadth measures used in the remaining of the manuscript have to be taken with caution, as acknowledged by the authors themselves. Consequently, the chance that results of this part of the work will enlighten the evolution of OR on a broad scale is low. For example, the fact that insect lineages that emerged after the Permian-Triassic extinction have more broadly-tuned OR is an interesting observation, yet remains highly speculative.

Thank you for this important comment. We agree that, even after optimizing the docking threshold, the current hit rate of approximately 20% remains insufficient to interpret molecular docking results as reliable predictions of actual OR ligand responses or functions. We also acknowledge that the biological implications of some docking results were still overstated in the previous revision, potentially leading readers to interpret these computational results as approximations of actual OR function. To avoid this misunderstanding, we have further revised the relevant terminology and interpretation of results throughout the latest manuscript. For results derived entirely from molecular docking, we no longer use terms such as ‘docking-derived function’, ‘functional community’, or other expressions that might imply experimental evidence of function. Instead, we consistently use ‘docking profile’ to make clear that these analyses reflect only similarities in computational docking patterns among receptors.

In addition, following your comments and the related suggestions from Reviewer 2, we have removed all results and discussion that directly linked docking-based predictions of OR function or recognition breadth to species ecology. We have explicitly presented the few remaining macroevolutionary comparisons as hypothesis-generating observations rather than evidence of functional evolution. For example, differences in docking-profile composition among extant insect lineages that originated before or after the end-Permian mass extinction (EPME) are now described only as a broad statistical pattern, with any interpretation framed as an experimentally untested hypothesis. Whether this pattern reflects actual differences in receptor tuning, and whether it is evolutionarily linked to environmental changes associated with the EPME, still requires testing through broader taxonomic sampling, reliable ancestral-state reconstruction, and functional experiments. We hope that these revisions restrict the interpretation of molecular docking results to what the evidence can support and bring the corresponding conclusions into closer alignment with the current strength of evidence.

The following revision has been added in Result section (lines 367-369): “These observations should be interpreted as a docking-derived comparative pattern among extant lineages rather than direct evidence of ancestral functional changes across the

EPME.”

And Discussion section (lines 410-414): “However, this interpretation remains hypothetical. Because rapid OR evolution and limited cross-order orthology preclude reliable reconstruction of deep ancestral repertoires, the EPME-related pattern should be viewed as a descriptive comparison among extant lineages. Further validation will require broader taxon sampling, ancestral reconstruction, and experimental testing of receptor tuning breadth.”

In summary, despite the large number of analyses performed, the authors do not really succeed in achieving the stated objective of reconstructing the evolutionary history of insect ORs, and the results obtained do not strongly support all the conclusions regarding the links between OR repertoires and environment or lifestyle.

Thank you for this important comment. We agree that ‘reconstructing the evolutionary history of insect ORs’ was too broad a formulation and exceeded what our methods could reliably address. A rigorous reconstruction of evolutionary history requires well-resolved phylogenetic relationships across taxa, ancestral-state inference, and systematic analysis of gene duplication and loss events. We have therefore redefined the scientific objectives of this study. The revised manuscript no longer claims to have reconstructed the evolutionary history of insect ORs. Instead, it aims to systematically characterize insect OR diversity and classification patterns at the levels of sequence, structure, and docking profile, compare the correspondence among these levels, and describe differences in their distribution across insect taxa and broad macroevolutionary contexts.

We have also reassessed the strength of different types of evidence concerning the relationships between OR repertoires and environment or lifestyle. For relationships between OR repertoire size and ecological traits, we repeated the genome-based benchmarking. After rerunning the ecological correlation sensitivity analyses using the updated OR count dataset, the main statistical results remained consistent with the original analyses. We therefore consider the current data to continue to support statistical associations between OR counts and ecological traits, with a degree of robustness. By contrast, we have removed results and discussion directly linking predicted OR function or recognition breadth to species ecology, and no longer use terms such as ‘docking-derived function’ or ‘functional community’ that might imply experimental evidence of function. The remaining macroevolutionary comparisons of docking profiles, such as differences in docking-profile composition among groups considered in relation to the EPME, are treated only as hypothesis-generating comparative patterns, rather than direct evidence of receptor functional evolution. We explicitly state that these hypotheses require future testing through functional experiments and more reliable ancestral-state reconstruction.

In summary, we have redefined the study objectives and no longer describe this work as a reconstruction of insect OR evolutionary history. We have retained the statistical associations between OR counts and ecological traits that remained supported after reassessment, while removing results and discussion linking receptor function to ecology on the basis of molecular docking. We believe these revisions better align the scientific objectives and scope of the conclusions with the evidence provided by the available data and methods.

We have revised the final paragraph of the Introduction to clarify the scope and objectives of this study (lines 80-91): “In this study, we characterized large-scale patterns of OR diversification across 115 species spanning 16 insect orders. Using a framework that integrates network analysis, protein structure prediction, and molecular docking, we constructed sequence-similarity (SSN), structural-similarity (StSN), and docking-profile similarity networks (dPSN) to classify ORs from three distinct perspectives. We identified differences in OR sequence-community diversity between holometabolous and non-holometabolous insects, as well as differences in docking-profile community composition among extant lineages whose order-level origins fall on opposite sides of the end-Permian mass extinction (EPME). Across this class-wide sampling, OR repertoire size was associated with adult diet, larval diet, and habitat, but not with circadian rhythm. Moreover, the conserved β-sheet in the extracellular loop 2 (EL2) region of Orco, together with its specialized binding-pocket architecture, is associated with its reduced ligand-binding capacity and represents a prominent structural specialization of the co-receptor lineage.”

**Reviewer #2 (Public review):**

The remarkable evolvability of the olfactory system enables animals to rapidly adapt to dynamic and chemically complex environments. Over the past two decades, substantial effort has been devoted to uncovering the evolutionary principles that drive the diversification of odorant receptors (ORs), yielding key insights into the forces shaping their striking variability in both vertebrates and insects. In this manuscript, Zhang and colleagues analyze the OR repertoires of over 100 insect species, leveraging sequence and structural similarity to infer patterns of gene family evolution within this diverse and ecologically important clade. By integrating sequence-based and structure-based comparisons, their study builds on a compelling and recently emerging line of research made possible by the advent of AlphaFold, which has previously clarified the phylogenetic relationship between insect Ors and the gustatory receptor gene family and revealed the unexpectedly deep evolutionary origins of this ancient structural fold.

Applying this approach to a large set of ORs derived from species throughout the insect phylogeny, the authors confirm many previously reported patterns of OR evolution. More importantly, with their large-scale approach the authors found new structural features of different Or clades, resulting in intriguing testable hypotheses about Or evolution that have the potential to support future directions in studying this important gene family. For example, the authors identify a structural feature mostly unique to the OR co-receptor ORco, a beta-sheet in EL2, which they functionally show reduces odorant binding affinity - a key aspect of ORco, which does not bind ligands in the ancestral ligand-biding site. This is a particularly strong part of the manuscript, since the authors support their in silico-derived hypothesis with functional data.

We thank the reviewer for the positive assessment of this part of our work.

In an attempt to assess the relationship between sequence identity and structure on one hand and function on the other, the authors perform an in-silico structure prediction and chemical docking analysis. While not suitable to assess the actual function of individual receptors without functional testing, this strategy revealed potential receptor clades with differences in the number and type of docked chemicals. As a response to the previous reviews, the authors now changed their writing to refer to these results as 'docking-derived function' instead of just 'function'. While I appreciate that the authors changed their wording, this semantic change still can be read as referring to dockingderived predictions as conclusions of actual biological function. However, this is not the case. Any functional claims based solely on the docking approach need to be interpreted with caution, as in silico-only approaches are currently not well enough predictors for actual receptor function. Accordingly, I strongly recommend to remove the parts that connect receptor function to structure or ecology.

Thank you for highlighting this important issue again. We agree with your comments and acknowledge that the presentation of molecular docking results in the previous revision still overstated their biological implications. Following your suggestion, we have removed all results and discussion that directly linked receptor function to species ecology solely on the basis of molecular docking, to avoid interpreting computational predictions as actual biological functions. We then systematically reviewed and revised docking-related terminology throughout the manuscript. For results derived entirely from docking analyses, we no longer use ‘docking-derived function’, ‘functional community’, or other expressions that might imply experimental evidence of function. Instead, we consistently use ‘docking profile’ to clarify that these analyses reflect similarities in computational docking patterns among receptors, rather than experimentally validated functional categories.

Through these revisions, we hope to distinguish more clearly between computational docking profiles and experimentally validated receptor function, and to avoid biological interpretations of docking results that exceed the available evidence. We believe these changes bring the manuscript's conclusions into closer alignment with the strength of the evidence.

Assessing their docking analyses with respect to species ecology, the authors identify correlations suggesting that Or repertoires might follow predictable evolutionary trajectories broadly aligned with species ecology. These are interesting hypotheses. However, due to the inability to predict actual receptor function the author's claims about ecology-function relationships are not well supported. Accordingly, the authors should change the respective parts of the manuscript in way that it is clear these are currently untested hypotheses. This includes adjusting the title, since the work does not find any experimental support for ecological tuning.

Thank you for this important suggestion. We agree that the current molecular docking analyses cannot support direct conclusions about relationships between actual receptor function and species ecology. Following your suggestion, we have removed the results and discussion directly linking docking-based predictions of OR function to species ecology. For the remaining macroevolutionary comparisons, such as differences in docking-profile composition among groups considered in relation to the EPME, we explicitly state that these results represent only computational patterns observed in extant taxa. The evolutionary interpretations proposed from these patterns remain experimentally untested hypotheses and cannot be considered direct evidence of changes in actual receptor function.

In addition, following your suggestion, we have changed the manuscript title to ‘Classwide sequence, structural, and docking-profile diversity of insect odorant receptors and structural specialization of Orco’. The new title no longer emphasizes ‘ecological tuning’ or ‘olfactory perception’, but instead focuses on class-wide diversification of insect OR sequences, structures, and docking profiles, together with the experimentally supported structural specialization of Orco.

The following revision has been added in Result section (lines 367-369): “These observations should be interpreted as a docking-derived comparative pattern among extant lineages rather than direct evidence of ancestral functional changes across the EPME.”

And Discussion section (lines 410-414): “However, this interpretation remains hypothetical. Because rapid OR evolution and limited cross-order orthology preclude reliable reconstruction of deep ancestral repertoires, the EPME-related pattern should be viewed as a descriptive comparison among extant lineages. Further validation will require broader taxon sampling, ancestral reconstruction, and experimental testing of receptor tuning breadth.”

I believe that future studies combining functional testing with an explicit evo-eco framework could benefit from this work and the explicit hypotheses it generates. But the authors should be careful to not overstate their in-silico derived results.

Thank you for this suggestion. We have revised the manuscript accordingly.

**Recommendations for the authors:**

**Reviewer #1 (Recommendations for the authors):**

Below are my comments in response to the rebuttal letter. I thank the authors for their careful revision and detailed responses to the points raised in my review.

(1) OR annotation pipeline and correlation analyses

Previous comment: "One can note aberrant numbers of ORs for certain species (much lower than reality), which indicates that the pipeline probably did not function correctly for all genomes. In the absence of a validation step comparing the results with already known OR repertoires, it is difficult to estimate the overall quality of the data. [...] It calls into question certain results that rely on this total number, such as the correlation between the number of ORs and genome size, lifestyle, and diet."

The response made by the authors on that point is not particularly convincing. They compared their OR gene annotations with previous reports in the same species, but this comparison is irrelevant because the chosen reference OR repertoires are actually a mix of transcriptome and genome annotations (the OR counts for *Harmonia axyridis*, *Plutella xylostella*, *Pierus brassicae*, *Dendrolimus kikuchii*, *Carposina sasakii*, *Hermetia illucens*, *Chlorops oryzae* and *Propsilocerus akamusi* are from transcriptomes). As not all OR genes are expressed in a given tissue, the number of ORs in transcriptomes is always lower than in genomes. Furthermore, comparisons with entire OR gene repertoires essentially demonstrate that their annotation was of good quality for species in which OR sequences were already present in the query OR database (*Bombyx mori*, *Drosophila melanogaster*, *Anopheles gambiae*) but was of bad quality for species in which OR sequences were NOT in the query (*Diabrotica virgifera*). However, re-annotation of Diabrotica led to an OR repertoire of 78 complete genes, vs. 124 in the manually curated genome annotation (1/3rd of the genes are missing). So these supplementary analyses made by the authors are not particularly in favor of an overall good quality of OR gene repertoires in the >110 species studied. They have also repeated the correlation analyses using 'full' OR gene sets where available. This showed that the correlation of OR count with genome size is no longer valid, but the correlation with diet or lifestyle is. However, if a mix of gene numbers from transcriptomes and genomes was used (see my comment above), I am afraid that these novel correlation analyses are not relevant. In conclusion, I would disagree with the claim that "These new benchmark and sensitivity analyses directly strengthen the evidence supporting our dataset and its ecological interpretations".

Thank you for further highlighting this issue. As detailed in our response to the Public Review, we repeated the OR annotation benchmarking, excluded transcriptome-only repertoires, and restricted comparisons to genome-based reference datasets or genome-based datasets supported by transcriptomic evidence. The updated results show that, for most species with comparable reference data, the number of ORs identified in this study exceeds 70% of the previously reported number. We also explicitly acknowledge that repertoire size may still be underestimated in some species.

We also directly tested whether query database composition systematically affected the number of annotated ORs. No significant difference was detected between species with and without conspecific OR queries (P = 0.841; P = 0.413 after excluding *Anagrus nilaparvatae*). Thus, we currently find no evidence that conspecific queries systematically increase the final OR count.

In addition, we repeated the sensitivity analyses of ecological correlations using the updated OR count dataset. The main statistical results remained unchanged: OR counts were still significantly associated with adult diet, larval diet, and habitat, but not with circadian rhythm. We therefore consider these statistical associations between OR counts and ecological traits to be reasonably robust to the annotation differences currently detectable. Detailed results are provided in our response to the Public Review and in the revised supplementary text S1.

(2) sequence similarity-based clusters

Previous comment: "The authors attempted to categorize ORs in several ways, starting with the reconstruction of sequence similarity networks. The approach is interesting, but in the end, the results do not seem to be sufficiently exploited. [...] For example, clusters SeqC26 or SeqC28, which appear to be shared by many insects, are potentially very interesting. It might have been relevant to combine this similarity-based clustering approach with phylogenetic reconstructions within each shared cluster."

The authors have followed this suggestion and identified an orthogroup conserved across several insect orders, which is an interesting observation. However the phylogeny of this orthogroup should be provided in supplementary data, which does not seem to be the case.

We thank the reviewer for the positive assessment. The corresponding phylogenetic tree has been added to the supplementary data S6.

(3) structure-based clusters

Previous comment: "The clusters shared by several orders are not truly exploited, which does not provide new insight into the evolution of ORs"

The authors show an interesting feature for a cluster of ORs from several insect orders, with a large putative binding pocket. However, it is not clear whether they verified or not if this cluster also correspond to an orthogroup or if this shared feature (a large binding pocket) is a convergence. They also improved the discussion of their results on a cluster of fly ORs with a long intracellular domain, which is a good thing.

Thank you for this important question. To determine whether StrC23, a structural community shared across multiple insect orders, corresponds to a conserved orthogroup, we performed additional phylogenetic analyses of its OR sequences and identified orthology relationships within the community based on the gene tree. The results showed that StrC23 does not correspond to a single orthogroup spanning insect orders, but instead contains 156 distinct orthologous groups. Of these, 24 encompass more than 10 species. Among these 24 larger groups, one contains ORs from Hymenoptera, Diptera, and Trichoptera, and two contain ORs from both Lepidoptera and Trichoptera. The remaining groups show pronounced order specificity, including nine Hymenoptera-specific groups, eight Lepidoptera-specific groups, and four Diptera-specific groups.

These results indicate that the large putative binding pocket characteristic of StrC23 is not explained by a single, broadly conserved orthogroup spanning multiple orders, but is distributed across several phylogenetically independent OR lineages. We therefore consider that this shared structural feature cannot simply be interpreted as direct retention within a conserved orthologous lineage. It may reflect convergent evolution toward similar structural features in different OR lineages, or the retention of an older structural feature in multiple OR lineages that subsequently underwent substantial sequence divergence. The current data do not allow us to distinguish conclusively between these two possibilities. We have added the phylogenetic analysis of StrC23 to the revised manuscript to clarify the evolutionary relationships within this structural community.

The following revision has been added (lines 208-218): “To further investigate the evolutionary basis of this broadly shared structural feature, we examined the phylogenetic and orthology relationships among StrC23 receptors. StrC23 was divided into 156 orthologous groups, 24 of which contained ORs from more than 10 species. However, only a few of these larger groups spanned multiple insect orders: one included ORs from Hymenoptera, Diptera, and Trichoptera, while two included ORs from both Lepidoptera and Trichoptera (Supplementary Data S6). Thus, StrC23 does not represent a single conserved cross-order orthologous lineage, but instead comprises multiple phylogenetically distinct OR lineages. Its shared large-pocket architecture therefore cannot be explained solely by conservation of a single orthologous lineage and may reflect either structural convergence or retention of an ancient structural feature followed by substantial sequence divergence.”

(4) molecular dynamics simulation

Previous comment: "The molecular dynamics modelling does not add anything in the way it is conducted (5 ns is too short)."

The authors argue that such a short simulation is sufficient for testing the interaction between a compound and the extracellular beta-sheet of the coreceptor Orco. I'm not a specialist enough to confirm whether this is actually true, but I trust the author's expertise in this area.

We thank the reviewer for the positive assessment.

(5 / 6) molecular docking and functional extrapolation

Previous comment: "in the absence of functional validation, this 20% true positive rate is not sufficient to extrapolate OR function"

The authors argue that molecular docking was not used to identify specific OR/ligand pairs, but rather to "estimate the potential binding tendencies of OR repertoires". I agree with this point; correlations were made using "binding breadth index values" that are calculated for entire OR repertoires. The text of the manuscript has been edited to refer to "docking-derived" functional clusters and binding breadth index, and to emphasise that the results should be interpreted with caution, which is a good point as it will prevent potential misinterpretation by the reader. However, this does not change the fact that interpreting the olfactory capacity of one species based on docking-derived binding breadth values of its OR repertoire is highly speculative (especially when OR repertoires are incomplete) and the chance that results of this part of the work will enlighten the evolution of OR on a broad scale is low.

Thank you for raising this concern. We agree that repertoire-level analyses do not resolve the inherent limitations of molecular docking in predicting actual receptor function. Although such analyses summarize overall docking patterns rather than individual OR–ligand interactions, they still cannot reliably define a species’ true olfactory capabilities. It therefore remains insufficient to describe a species' actual olfactory capabilities solely on the basis of docking-derived binding breadth. As detailed in our response to the Public Review, we have addressed this issue by following your suggestions and those of Reviewer 2 and removing the results and discussion that used the binding breadth index to directly link predicted receptor function to ecological traits such as diet, habitat, and circadian rhythm. We have also further standardized the terminology, replacing ‘docking-derived function’, ‘functional community’, and other expressions that could imply experimentally established function with ‘docking profile’, to emphasize that these results describe computational docking patterns rather than experimentally determined receptor functions. Through these revisions, we hope to distinguish more clearly between computational docking patterns and experimentally established receptor functions, and to restrict the interpretation of the related macroevolutionary comparisons to what the current data can support.

(7) EPME

Previous comment: "ORs of non-holometabolous insects cannot be considered equivalent to those of species that existed before the Permian-Triassic extinction"

The authors have now clarified the fact that they compared extant insect orders rather than ancestral and derived OR repertoires.

We thank the reviewer for the positive assessment of the revised manuscript.

(8 to 16)

The authors have carefully considered all the points raised in the recommendations for the authors, improving the manuscript on several aspects.

We thank the reviewer for the positive assessment of the revised manuscript.

Minor comment

Line 217: Orco did not emerge in the ancestor of Zygentoma, it emerged in the common ancestor of Zygentoma and Pterygota.

Thank you for pointing this out. We have corrected the statement.

**Reviewer #2 (Recommendations for the authors):**

The authors have done a good job in their revision of the manuscript. They addressed many of the reviewers' concerns well.

We thank the reviewer for the positive assessment of our previous revision.

However, the functional claims are still too strong and need to be removed or tempered down as described in the public review.

Thank you for this suggestion. We have removed or tempered the functional predictions in the manuscript in accordance with the Public Review. Please see our response to the Public Review for details.

Further, the manuscript title is not a good fit for the content. I would argue that the paper does not 'reveal ecological tuning shaping olfactory perception', since Or structurefunction relationships are involved in olfactory sensation or detection. Perception, however, requires interpretation, which is done by neural circuits but not receptors expressed within. Further, I still think that referring to 'ecological tuning' is far-fetched based on the docking approach that cannot really resolve the tuning of receptor sets. I would think that the work reveals interesting correlations between docking-derived population tuning and ecology. But that does not necessarily have anything to do with actual function and thus may not make much sense to advertise in the title or abstract.

Thank you for your suggestions concerning the manuscript title. Following this comment, we have changed the title from ‘Evolutionary dynamics of insect odorant receptors reveal ecological tuning shaping olfactory perception’ to ‘Class-wide sequence, structural, and docking-profile diversity of insect odorant receptors and structural specialization of Orco’ The new title no longer emphasizes ecological tuning or olfactory perception, but instead focuses on the two aspects of this study supported by the most direct evidence: class-wide patterns of sequence and structural diversification in insect ORs, and the experimentally supported structural specialization of Orco.

Consistent with the revised title, we have further adjusted the relevant wording in the Abstract, Results, and Discussion, and removed content directly linking receptor function to species ecology on the basis of docking results. We believe that the revised title and abstract more accurately reflect the main content of the study and the scope of its evidence, while avoiding functional or ecological interpretations of computational predictions that exceed their evidential support.

I suggest selecting a title that fits the sequence/structure/in silico docking nature of their large macroevolutionary approach and findings better. After all, apart from the orco work this manuscript is mostly a resource of in silico predictions.

Thank you for this suggestion. We have revised the manuscript title.

**Author response table 1.** Numbers of OR genes in previously annotated species.

Species	Annotation Method in Previous Study	Previous Reported OR Count	OR Count in This Study	Conspecific Query Available	Reference
Pieris brassicae	Genome + transcriptome	60	53	No	(Wang et al. 2023)
Plutella xylostella	Genome	95 (67 intact genes + 26 truncated genes + 2 pseudogenes)	77	Yes	(Engsontia et al. 2014)
Diabrotica virgifera virgifera	Genome + transcriptome	193 (124 intact genes)	78	No	(Paddock and Corcoran 2025)
Anagrus nilaparvatae	Genome	88	330	No	(Ma et al. 2022)
Solenopsis invicta	Genome	396	473	Yes	(Mier et al. 2022)
Gonatopus flavifemur	Genome	54	38	No	(Xiao et al. 2023)
Telenomus remus	Genome	46	57	No	(Xiao et al. 2023)
*Drosophila melanogaster*	Genome	60	56	Yes	(Robertson et al. 2003)
Bombyx mori	Genome	66 (including 2 pseudogenes)	64	Yes	(Tanaka et al. 2009)
Anopheles gambiae	Genome	79	67	Yes	(Carey et al. 2010)

Reference

Carey AF, Wang G, Su CY, Zwiebel LJ, Carlson JR. 2010. Odorant reception in the malaria mosquito Anopheles gambiae. Nature 464: 66-71.

Engsontia P, Sangket U, Chotigeat W, Satasook C. 2014. Molecular evolution of the odorant and gustatory receptor genes in lepidopteran insects: implications for their adaptation and speciation. J Mol Evol 79: 21-39.

Ma Y, Guo Z, Wang L, Wang B, Huang T, Tang B, Zhang G, Zhou Q. 2022. The genome of the rice planthopper egg parasitoid wasp Anagrus nilaparvatae casts light on the chemo- and mechanosensation in parasitism. BMC Genomics 23: 541.

Mier P, Fontaine JF, Stoldt M, Libbrecht R, Martelli C, Foitzik S, Andrade-Navarro MA. 2022. Annotation and Analysis of 3902 Odorant Receptor Protein Sequences from 21 Insect Species Provide Insights into the Evolution of Odorant Receptor Gene Families in Solitary and Social Insects. Genes (Basel) 13.

Paddock KJ, Corcoran JA. 2025. Life-stage dependent behavior mimics chemosensory repertoire diversity in a belowground, specialist herbivore. G3 (Bethesda) 15.

Robertson HM, Warr CG, Carlson JR. 2003. Molecular evolution of the insect chemoreceptor gene superfamily in *Drosophila melanogaster*. Proc Natl Acad Sci U S A 100 Suppl 2: 14537-14542.

Tanaka K, Uda Y, Ono Y, Nakagawa T, Suwa M, Yamaoka R, Touhara K. 2009. Highly selective tuning of a silkworm olfactory receptor to a key mulberry leaf volatile. Curr Biol 19: 881-890.

Wang Q, Dicke M, Haverkamp A. 2023. Sympatric Pieris butterfly species exhibit a high conservation of chemoreceptors. Front Cell Neurosci 17: 1155405.

Xiao S, Ye X, Wang S, Yang Y, Fang Q, Wang F, Ye G. 2023. Genome assembly of the ectoparasitoid wasp Theocolax elegans. Sci Data 10: 159.
